# Seasonal and landform effects on plant productivity and carrying capacity at Bisha Rangeland, Saudi Arabia

**DOI:** 10.3389/fpls.2026.1965543

**Published:** 2026-09-18

**Authors:** Abdullah S. Alharthi, Meshal A. Harbi, Sarah A. Almoghram, Ahmad Atmeh, Abdelhamid Elnaggar, Tamer Ismail, Eltohamy Yousef

**Affiliations:** 1 National Center for Vegetation Cover Development and Combating Desertification (NCVC), Riyadh, Saudi Arabia; 2 International Union for Conservation of Nature - Regional Office for West Asia (IUCN ROWA), Amman, Jordan; 3 Tilad Business Holding, Riyadh, Saudi Arabia

**Keywords:** adaptive rangeland management, arid rangelands, grazing management, topographic heterogeneity, vegetation productivity

## Abstract

Arid rangelands in Saudi Arabia face major management challenges due to pronounced seasonal variability and landform-related differences in vegetation productivity, necessitating science-based grazing strategies. However, the combined effects of these factors on plant biomass and livestock carrying capacity remain poorly quantified. To address this gap, this study investigated the individual and interactive effects of season and landform on vegetation productivity and livestock carrying capacity at Bisha-1 rangeland across four survey seasons (Autumn 2023, Spring 2024, Autumn 2024, and Spring 2025) and two landforms (valleys and plains). Highly significant differences were recorded among seasons in total palatable biomass (*p* < 0.001), which ranged from 86.64 kg DM ha^−1^ in Autumn 2023 to 215.84 kg DM ha^−1^ in Spring 2024, representing a 2.5-fold variation. Significant differences were also recorded between landforms (*p* = 0.007), with valleys exhibiting 20% higher total palatable biomass than plains. However, the season × landform interaction was non-significant (*p* = 0.810), indicating that seasonal patterns remained consistent across landform types. Palatable tree biomass was the dominant component, accounting for 70–80% of total palatable biomass across all season–landform combinations, whereas palatable shrub biomass made a smaller but seasonally variable contribution. Based on a standardized 90-day seasonal grazing model, livestock carrying capacity varied with season, ranging from 0.48 sheep units ha^-1–^90 days^-1^ in Autumn 2023 to 0.72 sheep units ha^-1–^90 days^-1^ in Spring 2024, representing a 1.5-fold variation, while landform effects on carrying capacity were secondary (valleys: 0.61 sheep units ha^-1–^90 days^-1^; plains: 0.53 sheep units ha^-1–^90 days^-1^) across the four seasons. These findings support seasonally adjusted stocking strategies, the conservation of palatable herbaceous and shrub components during periods of forage scarcity, and consideration of precipitation variability to sustain rangeland productivity in the Aseer region. However, interpretation of the estimated carrying capacity is limited by the single-site, four-campaign design and the absence of direct measurements of forage quality and animal utilization.

## 1 Introduction

Rangelands cover 54% of the global land surface and represent the dominant landscape type in arid and semi-arid regions (UNEP, 2021). These ecosystems provide critical resources for livestock production, wildlife conservation, and biodiversity maintenance while supporting millions of people globally (Herrera Calvo, 2024). Within this global context, the Kingdom of Saudi Arabia exemplifies the critical importance of rangelands, where these ecosystems occupy approximately 75% of the total land area (175 million hectares) and support over 2,250 plant species (Collenette, 1999; Chaudhary, 2001). Historically, Saudi Arabian rangelands were managed through traditional systems, including the Hema system, which maintained ecosystem productivity and species composition (Zahran and Younes, 1990; Al-Rowaily, 1999). Recent evidence further indicates that Al-Hima provides locally adapted principles for sustainable, long-term rangeland management in Arabian drylands (Al-Tabini and Smith, 2026).

However, the breakdown of traditional community-based management frameworks has led to uncontrolled resource overexploitation and open-access grazing (Al-Rowaily, 1999; Louhaichi et al., 2022). Consequently, Saudi Arabian rangelands have experienced significant degradation over recent decades, characterized by reduced vegetation cover, loss of palatable species, soil erosion, and declining productivity (Barth, 1999; Al-Rowaily, 2003; Al-Rowaily et al., 2018; Eldridge et al., 2026). Managing this vast geographic coverage presents complex conservation challenges, as vulnerable arid and semi-arid rangelands experience institutional collapse and habitat degradation that compromise native plant assemblages, reflecting wider regional dryland dynamics (Alharthi et al., 2021; Ghulami, 2024).

Unorganized grazing patterns, including premature grazing and overgrazing, together with sand drift and off-road vehicle traffic, represent important threats to rangeland sustainability in the Kingdom (Al-Rowaily, 2003; Alzarah et al., 2021; Sayed et al., 2026). Specifically, premature grazing depletes plant root reserves and reduces seasonal biomass accumulation (Liu et al., 2016; Ren et al., 2017). Furthermore, continuous overgrazing imposes persistent pressure on vegetation without adequate recovery periods, resulting in systematic reduction of palatable species and altered species composition (Rahmanian et al., 2020; Abdelsalam, 2021; Gebremedhn et al., 2023; Biancari et al., 2024; Pichon et al., 2025). This landscape degradation is caused primarily by intense human disturbances that undermine rangeland stability, induce severe physiological stress, and erode genetic diversity within native plant populations, thereby weakening their capacity for natural regeneration (Al-Rowaily et al., 2018; Alhaithloul, 2019). Consequently, recovery from intensive overgrazing requires decades, demonstrating the long-term consequences of these degradation processes (Varga et al., 2021).

In response to these challenges, effective rangeland rehabilitation requires science-based management frameworks informed by accurate assessments of carrying capacity (Letsoalo et al., 2025). Such frameworks should be integrated with community-based approaches to promote local participation and long-term socio-ecological sustainability (Slayi, 2025). Carrying capacity is a dynamic ecological parameter that varies across space and time in relation to seasonal conditions, vegetation composition, and soil characteristics (Meshesha et al., 2019). In arid and semi-arid rangelands, plant productivity is strongly regulated by seasonal variation in precipitation and temperature, which determines the timing and magnitude of vegetative growth (Chen and Weber, 2014; Churchill et al., 2022; Schantz et al., 2023). Recent remote-sensing evidence from Saudi Arabia also shows pronounced seasonal and annual variation in vegetation dynamics associated with rainfall and temperature conditions (Assiri et al., 2025). During spring, rainfall commonly coincides with favorable temperatures and active growth of perennial species, thereby promoting biomass accumulation, vegetation cover, and reproductive activity (Chen and Weber, 2014; Rousta et al., 2024). In arid and semi-arid environments, precipitation pulses may generate short periods of rapid primary production, including increased leaf-area development and aboveground biomass (Post and Knapp, 2020; Liang et al., 2021). Favorable spring temperatures, increasing photoperiod, and adequate soil moisture further support photosynthesis and forage production. In contrast, reduced soil moisture, shorter photoperiods, and lower temperatures during autumn may constrain plant growth and reduce primary productivity. Previous monitoring studies have documented substantial intra-annual variation in phytomass between seasonal growth periods, with several-fold differences in biomass production in highly variable systems (Magandana et al., 2020; Lasché et al., 2023; Zhao et al., 2024). Accordingly, seasonal climatic variability should be incorporated into adaptive rangeland-management frameworks.

In addition to seasonal variations, landform characteristics and topographic heterogeneity are important drivers of spatial variation in plant biodiversity and productivity as they create microenvironments that influence vegetation distribution and ecosystem functioning across arid landscapes (Wei et al., 2019; Sanaei et al., 2019; Zhang et al., 2022; Yang et al., 2023; Li et al., 2023). In arid and semi-arid regions, local topography modifies the distribution of water, nutrients, and thermal conditions at fine spatial scales, thereby creating pronounced spatial variation in primary productivity and floristic composition (Peng et al., 2023; Luo et al., 2024). These effects are especially evident in dissected landscapes, where elevation, slope exposure, and drainage networks form environmental mosaics that support distinct plant assemblages and pastoral resource niches. Within this context, valleys (wadi systems) generally show higher productivity than adjacent plains because runoff concentration and sediment deposition enhance water and nutrient accumulation (Ali et al., 2000; El-Bana and Al-Mathnani, 2009; Elaidarous et al., 2022; Alqurashi and Alharbi, 2025). In contrast, plains tend to have more spatially uniform but lower productivity because of limited water accumulation and more extreme microclimatic conditions (Wei et al., 2019). Nevertheless, plains support plant functional groups adapted to xeric conditions and therefore contribute to landscape-scale diversity and ecosystem resilience (Biancari et al., 2024; de Oliveira et al., 2024).

Despite the recognized influence of seasonal variability and landform heterogeneity on rangeland productivity, field-based assessments that simultaneously quantify their main and interactive effects on herbaceous, shrub, and tree biomass and estimated livestock carrying capacity remain limited in Saudi Arabian rangelands. This knowledge gap significantly limits the development of adaptive management strategies that could optimize forage utilization while preventing rangeland degradation. Therefore, this research aimed to assess vegetation biomass production across different seasons and landforms at Bisha-1 rangeland site in the Aseer region, Kingdom of Saudi Arabia, and to quantify the individual and interactive effects of season and landform on rangeland productivity and estimated carrying capacity. To achieve these objectives, we tested three hypotheses: (i) palatable biomass and estimated carrying capacity would be higher during spring than autumn; (ii) valleys would have higher palatable biomass and estimated carrying capacity than plains; and (iii) the magnitude of seasonal variation in palatable biomass and estimated carrying capacity would differ between valleys and plains. These findings provide critical empirical data for developing dynamic, season-based grazing management strategies that address overgrazing challenges while ensuring long-term ecological health and sustainable utilization of these vital rangeland resources.

## 2 Materials and methods

### 2.1 Study area

This study was conducted at Bisha-1 rangeland site within Bisha Governorate, Aseer Region, southwestern Saudi Arabia (**Figure 1**). The site covers approximately 812.61 km² (81,261 hectares) and is located between 19.487–19.824° N and 42.685–42.975° E, approximately 21 km south of Bisha city, serving as a grazing resource for livestock owners in the surrounding villages. Elevation ranges from 1,260 to 1,510 m, with approximately 80% of the area between 1,310 to 1,460 m. Terrain slopes range from 0° to 15°, with 76% of the area below 7.5°. The majority of slopes face west and northwest. Soils are predominantly deep and silty-calcareous (86% of the area), while rock outcrops and mountains cover approximately 14%.

**Figure 1:**
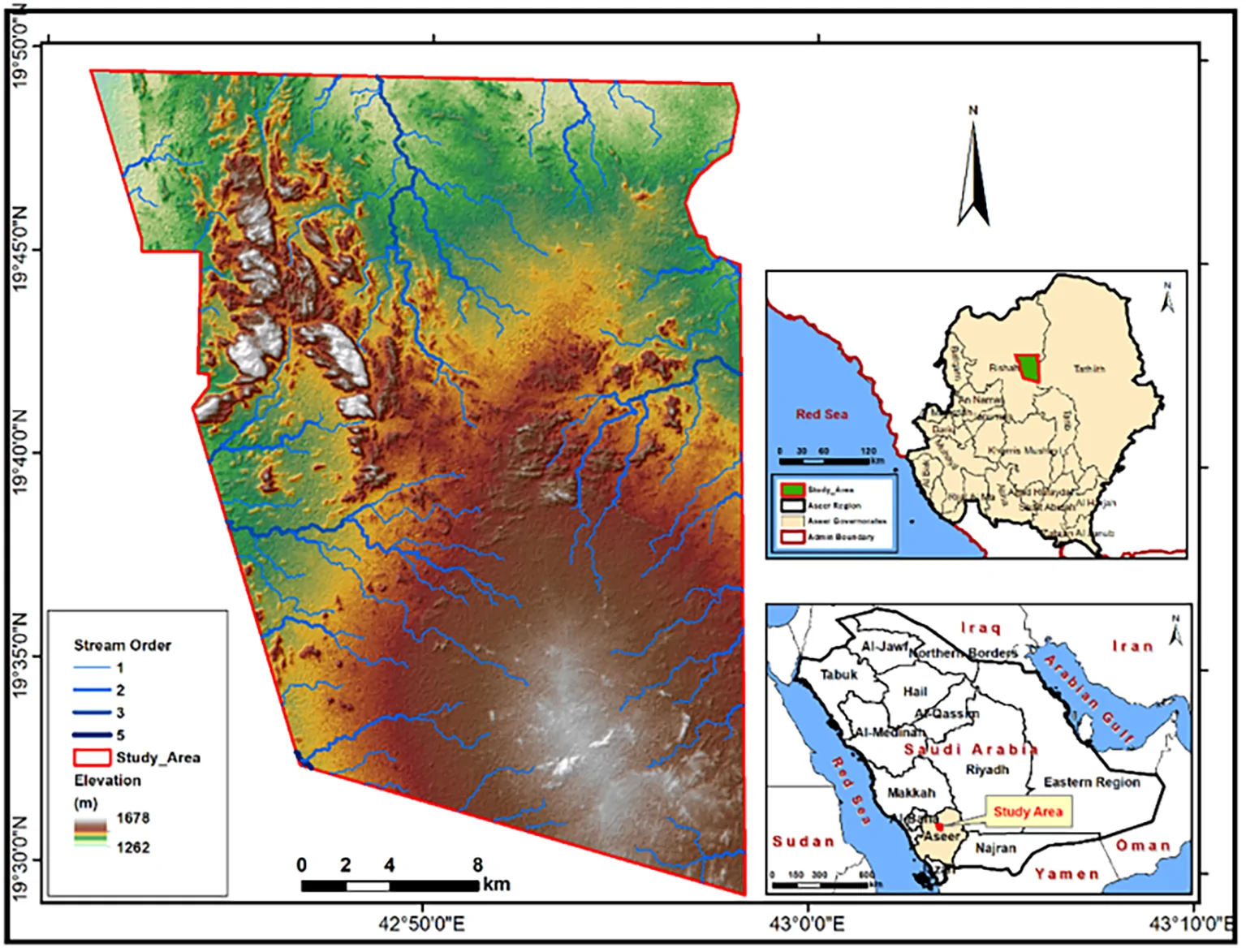
Geographic location and topographical features of Bisha-1 rangeland site in southwestern Saudi Arabia.

The primary landforms are plains and valleys surrounded by mountains (**Figure 2**). The hydrological network includes three main valley systems: Wadi Al-Khanq (extending north-central to west), Wadi Al-Hanthariyah (extending westward), and Wadi Harjah with its tributaries (extending southwest).

**Figure 2:**
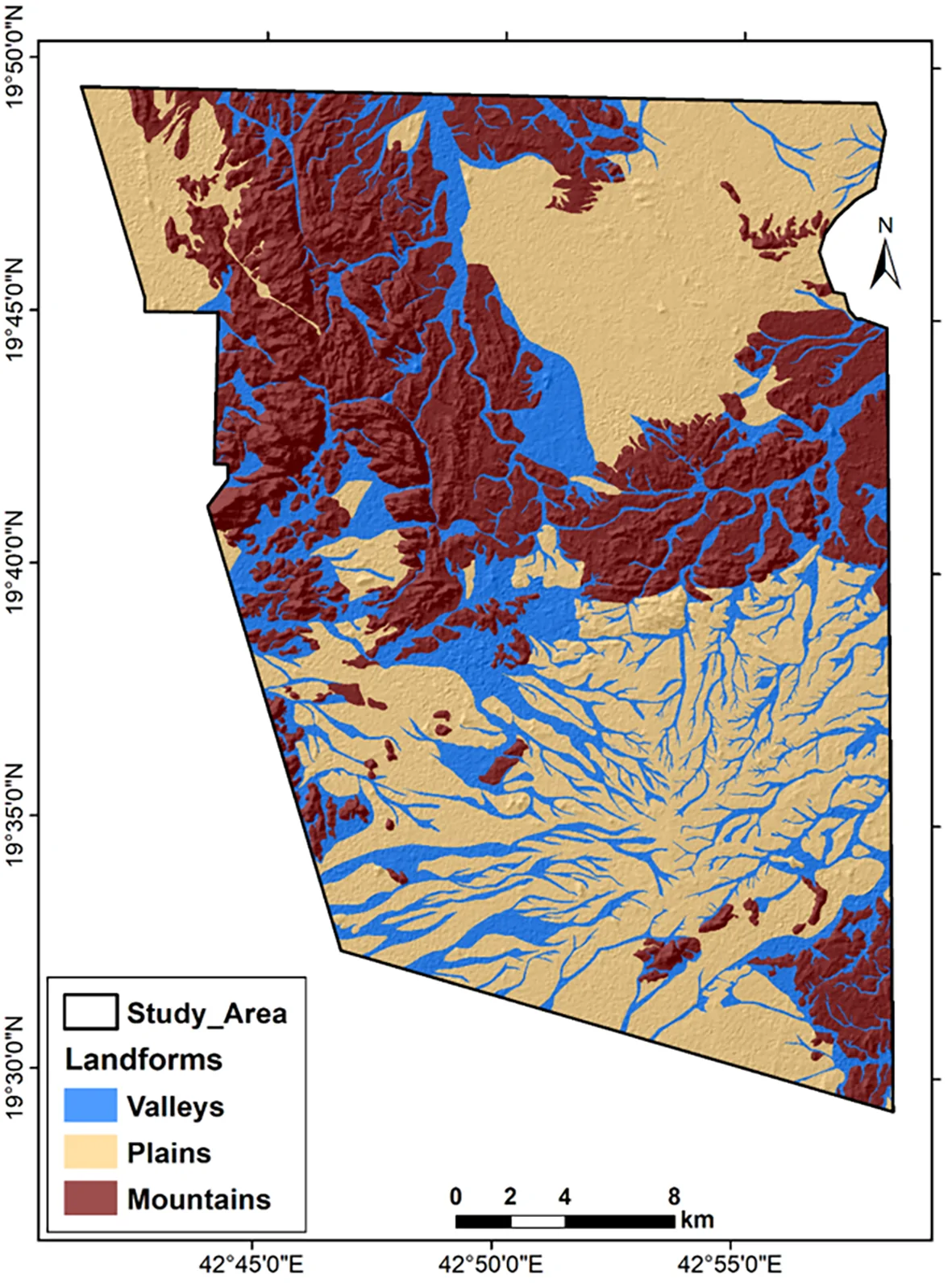
Distribution of main landforms (plains and valleys) within Bisha-1 rangeland site.

### 2.2 Sampling design and allocation

The study area was first stratified in a Geographic Information System (GIS) into two landform strata: valleys and plains. Subsequently, the Normalized Difference Vegetation Index (NDVI) was calculated from Sentinel-2 satellite imagery acquired in October 2023 using the Google Earth Engine environment. A median composite of cloud-free images was used to calculate NDVI according to the equation NDVI = (B8 − B4)/(B8 + B4), following Rouse et al. (1974), where B8 is the near-infrared band and B4 is the red band; both bands have a 10-m spatial resolution. Full details of the NDVI calculation procedure are provided in **Supplementary Data 1**. The NDVI map was used to identify the spatial distribution of vegetation within each landform stratum and to guide the initial allocation of sampling locations.

Local field-verification tours were conducted in October 2023 over three days across the studysite along locally used access routes, with the assistance of a local resident. These tours were used to verify NDVI-derived vegetation patterns, plant-community distribution, and plant density and to guide the selection of representative sampling points within valleys and plains. Based on the NDVI mapping and field verification, a total of 32 sampling points were selected, including 23 points in valleys and 9 points in plains. GPS coordinates of all sampling points were recorded (**Supplementary Table 1**).

### 2.3 Local livestock-owner interviews

Local livestock-owner interviews were conducted to document grazing practices and plant-use information for the Bisha-1 rangeland site. Official records listed 187 livestock owners from villages surrounding the study site. During the three-day ground-truthing tours, livestock owners were approached, and 56 owners who agreed to participate were interviewed, representing 29.9% of the officially registered owners. Information collected included the timing and duration of livestock grazing at the study site, commonly grazed plant species, local plant names, and the perceived palatability of these species. The mean grazing duration was calculated and used as the standardized 90-day grazing period in the carrying-capacity calculation.

### 2.4 Vegetation survey

Vegetation was sampled using systematic transect-quadrat methods to capture different plant growth forms. At each sampling point, three 100 m transects were established approximately 100 m apart. Woody vegetation (trees and shrubs) was sampled in 100 m² rectangular quadrats (5 m × 20 m), while herbaceous vegetation was sampled in 1 m² quadrats. Ten quadrats were positioned at 10-meter intervals along each transect line, alternating between right and left sides to minimize spatial autocorrelation. This design resulted in 30 replicates each for woody and herbaceous vegetation at each sampling point. Field surveys were conducted at four survey campaigns: Autumn 2023, Spring 2024, Autumn 2024, and Spring 2025 (**Table 1**).

**Table 1:** Vegetation survey schedule and sampling dates for seasonal biomass assessment at Bisha-1 rangeland site during the study period 2023-2025.

Season	Survey date
Autumn 2023	17-21/12/2023
Spring 2024	22-26/5/2024
Autumn 2024	12-17/11/2024
Spring 2025	9-13/3/2025

Within the quadrats, plant species were identified and classified following the taxonomicframework of Chaudhary (2013), with all recorded speciesdocumented in **Supplementary Table 2**. Palatability classifications were determined according to Al-Jaloud et al. (1994) and local livestock owner knowledge to ensure comprehensive and regionally accurate classification as mentioned above.

### 2.5 Biomass determination

#### 2.5.1 Herbaceous vegetation

All herbaceous vegetation within each 1 m² quadrat was harvested by clipping at 5 cm above the soil surface to simulate livestock grazing height. Fresh weight was immediately recorded using a portable electronic balance (± 0.1 g precision). Samples were placed in labeled paper bags and transported to the laboratory. Samples were dried in forced-air ovens at 70 °C until constant weight (typically 72 hours), and final dry weight was recorded to calculate dry matter content.

#### 2.5.2 Woody vegetation (shrubs and trees)

The reference-unit method (Andrew et al., 1976) was employed to estimate woody biomass. For each plant species, a representative individual was selected based on average size and growth characteristics. For shrub species, approximately 25% of the total plant volume was systematically clipped as the reference unit. For tree species, a reference branch approximately 5 cm in diameter was selected and clipped. Each reference unit was separated into wood and browse components, where browse represents the edible portion accessible to grazing animals. For tree species, only branches or portions of branches below 2.5 m above ground level were included in the browse biomass estimate; browses above this height were excluded because they were considered inaccessible to grazing animals. The number of equivalent reference units present on each plant was counted independently by two observers, and the mean count was used for biomass estimation. Fresh browse weight from the reference unit was measured immediately, followed by oven drying as described for herbaceous vegetation. Total browse biomass per plant was calculated by multiplying the dry browse weight of the reference unit by the mean number of equivalent reference units per plant.

### 2.6 Carrying capacity calculation

Carrying capacity (CC) was calculated using a systematic approach that considers total palatable biomass, utilization factors, daily livestock requirements and grazing period as follows:


$$\text{CC}=\frac{TPB\times UF}{DFR\times GP}$$

Where,

- CC is carrying capacity (sheep units ha^-1–^90 days^-1^);
- TPB is the total palatable biomass (kg DM ha^−1^);
- UF is utilization factor [30% for total palatable biomass; following recommendations by Meshesha et al. (2019) and Fenetahun et al. (2022)].
- DFR is daily feed requirement (2.5% of the assumed 40-kg sheep body weight, equivalent to 1.0 kg DM sheep unit^−1^ day^−1^);
- GP is the grazing period (90 days, based on field survey as mentioned above)

### 2.7 Climatic data

Climatic data (precipitation and temperature) were obtained from high-resolution remote sensingdatasets to provide consistent spatial and temporal coverage. Maximum and minimum temperatures wereextracted from TerraClimate, a high-resolution dataset (4 km spatial resolution) providing monthly climatic data. Precipitation data were derived from CHIRPS (Climate Hazards Group InfraRed Precipitation with Station data), which provides rainfall estimates at 0.05° spatial resolution (approximately 5.3 km). Climatic data were extracted from 1990-2025, and detailed seasonal and annual data are provided in **Supplementary Tables 3**–**5**.

### 2.8 Statistical analysis

All statistical analyses were performed using R Statistical Software (R version 4.4.0; R Core Team, 2024). Prior to analysis, data were tested for normality using the Shapiro-Wilk test and for homogeneity of variance using Levene’s test. Two-way analysis of variance (ANOVA) was conducted using the *aov* function in the stats R package to examine the main effects of season (Autumn 2023, Spring 2024, Autumn 2024, and Spring 2025) and landform (valley and plain), as well as their interaction, on nine response variables: palatable herbage biomass (kg DM ha^−1^), palatable shrub biomass (kg DM ha^−1^), palatable tree biomass (kg DM ha^−1^), total palatable biomass (kg DM ha^−1^), unpalatable herbage biomass (kg DM ha^−1^), unpalatable shrub biomass (kg DM ha^−1^), unpalatable tree biomass (kg DM ha^−1^), total unpalatable biomass (kg DM ha^−1^), and carrying capacity (sheep units ha^-1–^90 days^-1^).

Due to the unbalanced sampling design across landforms (N = 9 for plains; N = 23 for valleys), model-derived Estimated Marginal Means (EMMeans) were calculated to obtain model-adjusted means under the unequal sampling allocation. *Post-hoc* pairwise comparisons of treatment means were conducted using Tukey’s Honest Significant Difference (HSD) test with a family-wise error rate of α = 0.05. To assess the precision of landform comparisons under the unequal sampling allocation, *post-hoc* power was calculated for the Landform main effect in each two-way ANOVA at α = 0.05, using the observed F statistic and its associated degrees of freedom.

Principal Component Analysis (PCA) was performed using the *prcomp* function on nine standardized variables to examine multivariate relationships among biomass components across seasons and landforms. All variables were standardized using z-score transformation prior to analysis. Principal components were extracted based on eigenvalues, and loadings were interpreted to identify the contribution of each variable to the principal components. A biplot was constructed displaying observations color-coded by season and shape-coded by landform, with 95% confidence ellipses overlaid for each season.

## 3 Results

### 3.1 Analysis of variance

Two-way ANOVA revealed that season was the primary driver of variation in biomass components (**Table 2**). Specifically, season exhibited highly significant effects on all palatable biomass components (herbaceous, shrub, and tree biomass) and total palatable biomass (all *p<* 0.001), as well as unpalatable herbaceous biomass (*p* = 0.005) and livestock carrying capacity (*p* = 0.002). Conversely, season had no significant effect on unpalatable shrub biomass (*p* = 0.270), unpalatable tree biomass (*p* = 0.371), or total unpalatable biomass (*p* = 0.401).

**Table 2:** Analysis of variance (ANOVA) for biomass components and carrying capacity by season and landform at Bisha-1. .

Variable	Source of Variation	df	F-value	p-value
Palatable herbage biomass (Kg DM ha^−1^)	Season	3	18.159	< 0.001
	Landform	1	3.148	0.079
	Season × Landform	3	2.365	0.074
Palatable shrub biomass (Kg DM ha^−1^)	Season	3	11.552	< 0.001
	Landform	1	0.015	0.904
	Season × Landform	3	0.520	0.669
Palatable tree biomass (Kg DM ha^−1^)	Season	3	24.246	< 0.001
	Landform	1	14.218	< 0.001
	Season × Landform	3	0.379	0.768
Total palatable biomass (Kg DM ha^−1^)	Season	3	31.423	< 0.001
	Landform	1	7.505	0.007
	Season × Landform	3	0.321	0.810
Unpalatable herbage biomass (Kg DM ha^−1^)	Season	3	4.528	0.005
	Landform	1	4.651	0.033
	Season × Landform	3	0.984	0.403
Unpalatable shrub biomass (Kg DM ha^−1^)	Season	3	1.324	0.270
	Landform	1	16.500	< 0.001
	Season × Landform	3	0.390	0.760
Unpalatable tree biomass (Kg DM ha^−1^)	Season	3	1.055	0.371
	Landform	1	2.231	0.138
	Season × Landform	3	0.432	0.730
Total unpalatable biomass (Kg DM ha^−1^)	Season	3	0.988	0.401
	Landform	1	12.708	< 0.001
	Season × Landform	3	0.851	0.469
Carrying capacity (sheep units ha^-1–^90 days^-1^)	Season	3	5.247	0.002
	Landform	1	2.310	0.131
	Season × Landform	3	0.091	0.965

Regarding landform effects, they were component-specific and generally less pronounced than seasonal effects. Specifically, landform significantly influenced palatable tree biomass (*p* < 0.001), total palatable biomass (*p* = 0.007), unpalatable herbaceous biomass (*p* = 0.033), unpalatable shrub biomass (*p* < 0.001), and total unpalatable biomass (*p* < 0.001) (**Table 2**). However, landform had no significant effect on palatable herbaceous biomass(*p* = 0.079), palatable shrub biomass(*p* = 0.904), unpalatable tree biomass (*p* = 0.138), or carrying capacity (*p* = 0.131). *Post-hoc* power analysis for the Landform main effect ranged from 0.052 to 0.982 across the response variables (**Supplementary Table 6**). Power was high for the significant landform effects on palatable tree biomass (0.965), total unpalatable biomass (0.946), and unpalatable shrub biomass (0.982), and moderate for total palatable biomass (0.782). In contrast, power for the non-significant landform effect on carrying capacity was low (0.330), indicating limited precision for detecting small differences between valleys and plains.

Moreover, the interaction between season and landform was non-significant for all biomass components and carrying capacity (all *p* ≥ 0.074), indicating that seasonal patterns were consistent across landform types throughout the study period.

### 3.2 Main effect of season

Seasonal variation significantly influenced palatable biomass components and estimated livestock carrying capacity, with generally greater productivity during the spring seasons than during the autumn seasons (**Figure 3**). Across the four survey campaigns, palatable herbaceous biomass ranged from 0.98 to 24.55 kg DM ha^−1^, with the highest value recorded in Spring 2024 and the lowest in Autumn 2023. Palatable shrub biomass varied from 5.65 kg DM ha^−1^ in Autumn 2024 to 19.90 kg DM ha^−1^ in Spring 2024. Similarly, palatable tree biomass was lowest in Autumn 2023 (78.93 kg DM ha^−1^) and highest in Spring 2024 (171.39 kg DM ha^−1^). As a result, total palatable biomass ranged from 86.64 kg DM ha^−1^ in Autumn 2023 to 215.84 kg DM ha^−1^ in Spring 2024.

**Figure 3:**
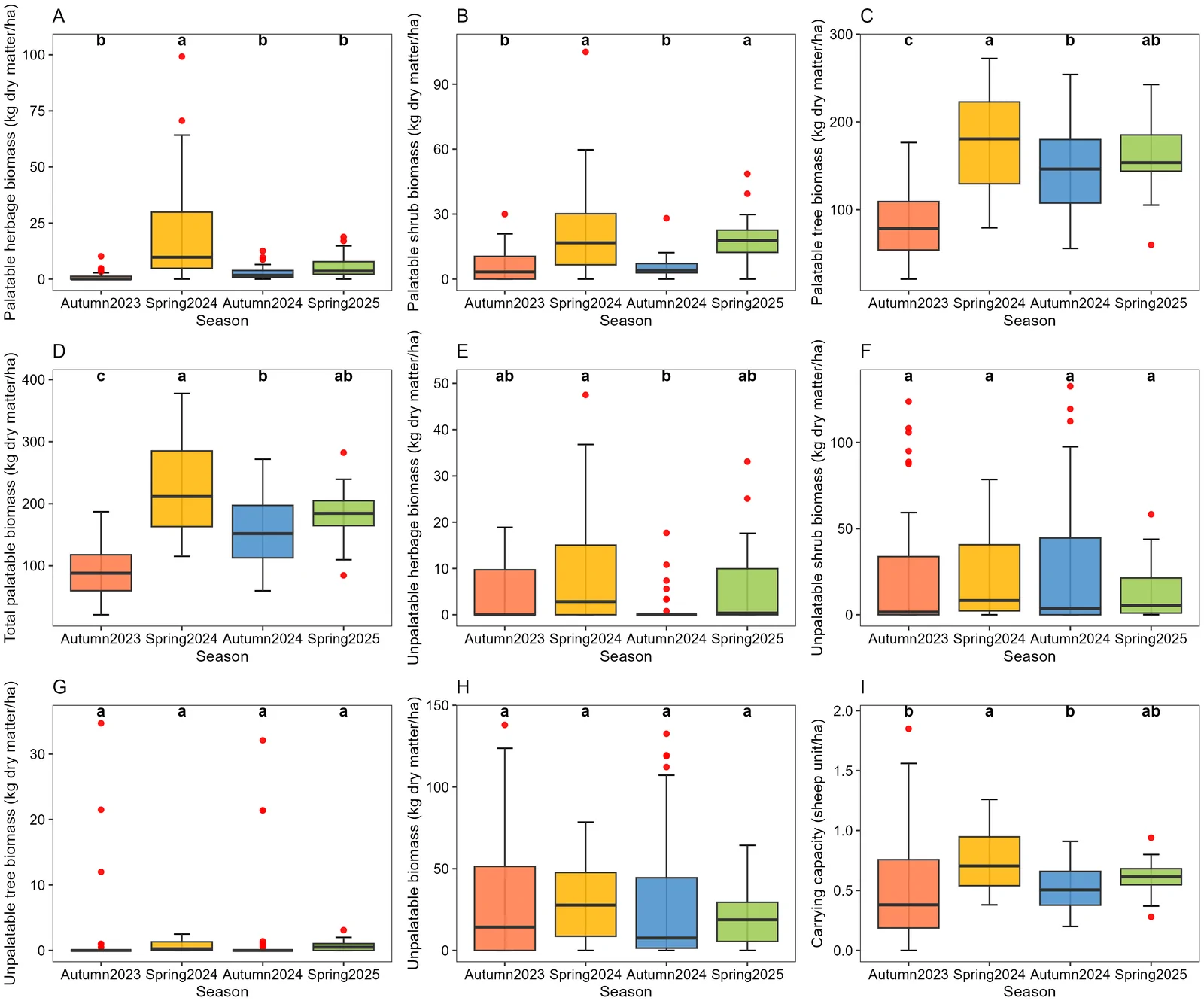
Seasonal effect on biomass components and carrying capacity at Bisha-1 rangeland site across four consecutive seasons (Autumn 2023, Spring 2024, Autumn 2024, and Spring 2025). **(A)** palatable herbage biomass, **(B)** palatable shrub biomass, **(C)** palatable tree biomass, **(D)** total palatable biomass, **(E)** unpalatable herbage biomass, **(F)** unpalatable shrub biomass, **(G)** unpalatable tree biomass, **(H)** total unpalatable biomass, and **(I)** carrying capacity. Different letters above bars indicate significant differences at P< 0.05 (Tukey’s HSD test).

In contrast, seasonal variation in unpalatable biomass was limited (**Figure 3**). Unpalatable herbaceous biomass ranged from 1.62 kg DM ha^−1^ in Autumn 2024 to 9.78 kg DM ha^−1^ in Spring 2024 and differed significantly among seasons. However, unpalatable shrub biomass showed no significant seasonal difference, despite ranging from 10.36 kg DM ha^−1^ in Spring 2025 to 20.85 kg DM ha^−1^ in Autumn 2024. The values for unpalatable tree biomass were similarly low across seasons, ranging from 0.56 kg DM ha^−1^ in Spring 2025 to 1.84 kg DM ha^−1^ in Autumn 2024. Consequently, total unpalatable biomass did not differ significantly among seasons and ranged from 18.52 kg DM ha^−1^ in Spring 2025 to 26.80 kg DM ha^−1^ in Autumn 2023.

Estimated livestock carrying capacity followed the overall pattern of total palatable biomass. It ranged from 0.48 sheep units ha^−1^ per 90-day grazing period in Autumn 2023 to 0.72 sheep units ha^−1^ per 90-day grazing period in Spring 2024 (**Figure 3**; **Table 2**).

### 3.3 Main effect of landform

Palatable herbaceous and shrub biomass showed no significant differences between landforms, with Plain and Valley averaging 10.75 and 6.48 kg DM ha^−1^ for herbaceous biomass, and 12.83 and 12.52 kg DM ha^−1^ for shrub biomass, respectively (**Figure 4**). However, Valley exhibited significantly higher palatable tree biomass (152.61 kg DM ha^−1^) compared to Plain (118.87 kg DM ha^−1^), with a difference of 33.74 kg DM ha^−1^. Consequently, total palatable biomass was significantly higher in Valley (171.62 kg DM ha^−1^) than Plain (142.44 kg DM ha^−1^), representing a difference of 29.18 kg DM ha^−1^.

**Figure 4:**
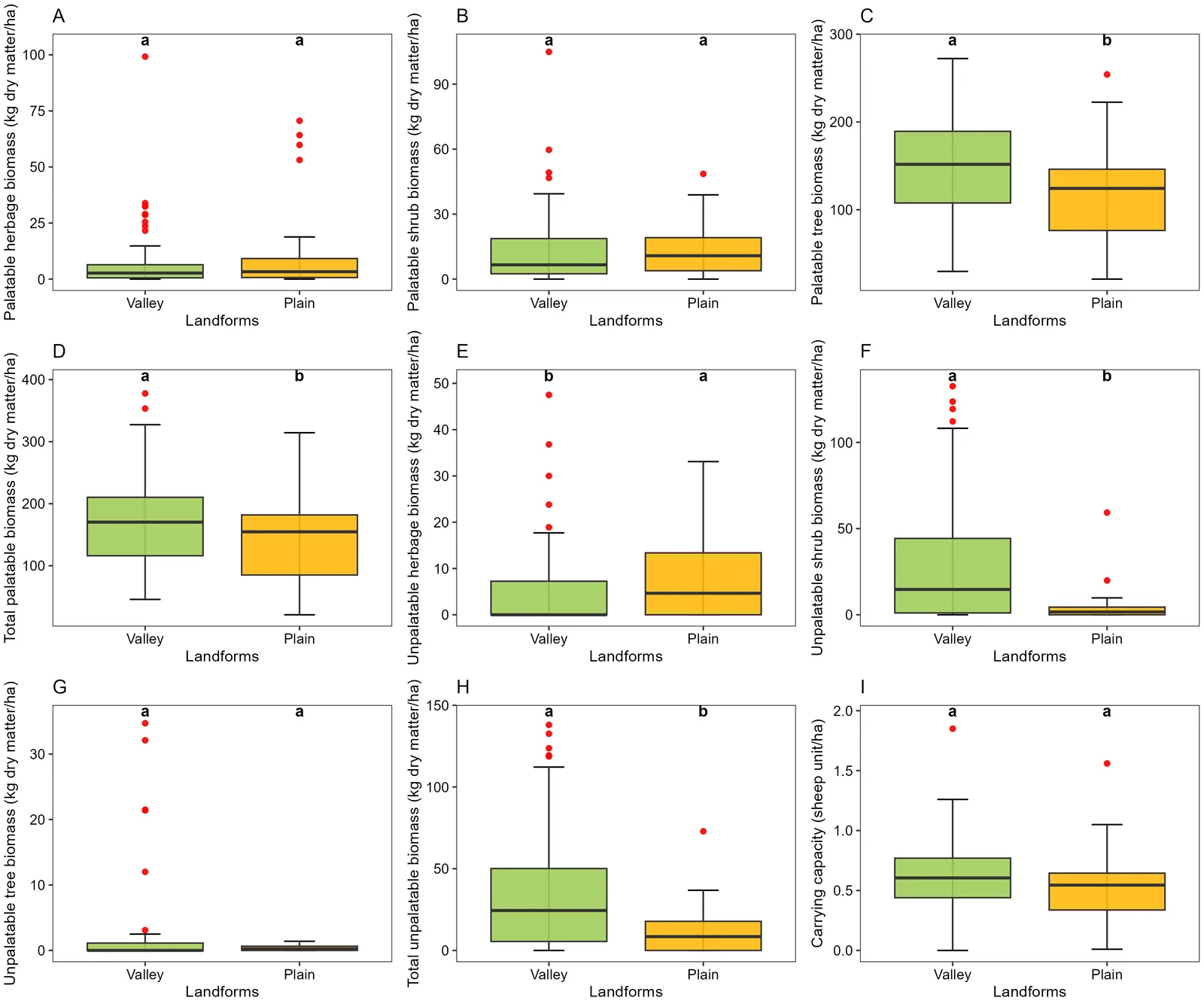
Landform effect on biomass components and grazing capacity: **(A)** palatable herbage biomass, **(B)** palatable shrub biomass, **(C)** palatable tree biomass, **(D)** total palatable biomass, **(E)** unpalatable herbage biomass, **(F)** unpalatable shrub biomass, **(G)** unpalatable tree biomass, **(H)** total unpalatable biomass, and **(I)** carrying capacity. Different letters above bars indicate significant differences at P< 0.05 (Tukey’s HSD test).

Unpalatable biomass components demonstrated variable landform responses. Plain exhibited significantly higher unpalatable herbage biomass (7.81 kg DM ha^−1^) compared to Valley (4.30 kg DM ha^−1^), with a difference of 3.51 kg DM ha^−1^. In contrast, Valley demonstrated significantly higher unpalatable shrub biomass (29.05 kg DM ha^−1^) than Plain (4.45 kg DM ha^−1^), representing a difference of 24.60 kg DM ha^−1^. Unpalatable tree biomass showed no significant differences between landforms, with Valley averaging 1.95 kg DM ha^−1^and Plain averaging 0.40 kg DM ha^−1^. Total unpalatable biomass was significantly higher in Valley (35.33 kg ha^−1^) compared to Plain (12.68 kg DM ha^−1^), with a difference of 22.65 kg DM ha^−1^.

Livestock carrying capacity showed no significant main effect of landform, with values of 0.61 and 0.53 sheep units ha^-1–^90 days^-1^ in Valley and Plain, respectively.

### 3.4 Interaction effect (season × landform)

Across the individual season–landform combinations, total palatable biomass ranged from 67.24 kg DM ha^−1^ on the plain in Autumn 2023 to 231.42 kg DM ha^−1^ in the valley in Spring 2024. Similarly, palatable herbaceous biomass was highest on the plain in Spring 2024 (32.06 kg DM ha^−1^) and lowest on the plain in Autumn 2023 (0.56 kg DM ha^−1^). Palatable shrub biomass also varied, ranging from 5.14 kg DM ha^−1^ in the valley in Autumn 2023 to 22.31 kg DM ha^−1^ in the valley in Spring 2024. In the same way, palatable tree biomass ranged from 58.38 kg DM ha^−1^ on the plain in Autumn 2023 to 192.08 kg DM ha^−1^ in the valley in Spring 2024 (**Figure 5**).

**Figure 5:**
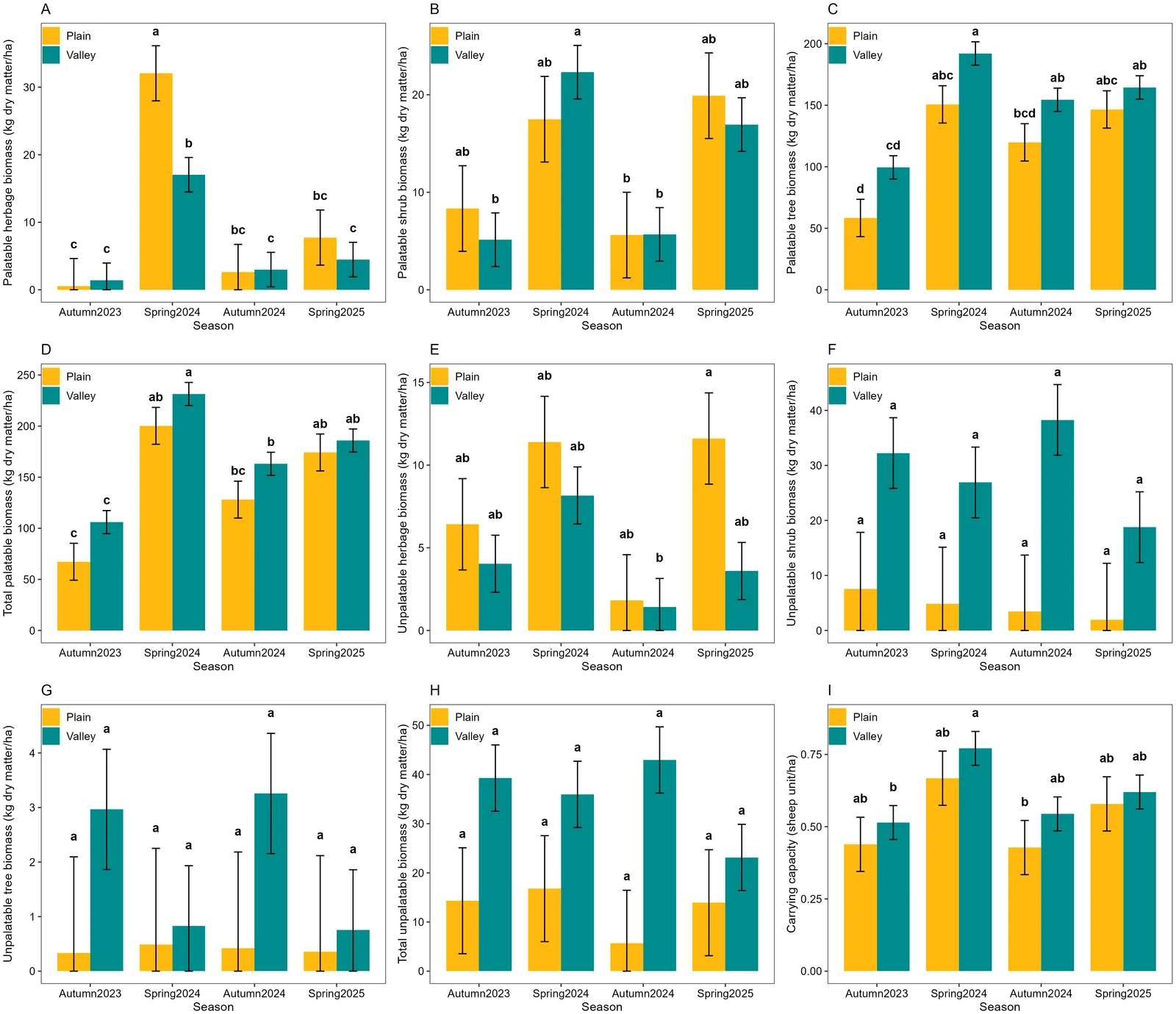
Interaction effect of season and landform on biomass components and carrying capacity at Bisha-1 rangeland site: **(A)** palatable herbage biomass, **(B)** palatable shrub biomass, **(C)** palatable tree biomass, **(D)** total palatable biomass, **(E)** unpalatable herbage biomass, **(F)** unpalatable shrub biomass, **(G)** unpalatable tree biomass, **(H)** total unpalatable biomass, and **(I)** carrying capacity. Error bars represent standard error (SE). Different letters above bars indicate significant differences at P< 0.05 (Tukey’s HSD test).

With respect to unpalatable biomass, herbaceous biomass ranged from 1.42 kg DM ha^−1^ in the valley in Autumn 2024 to 11.61 kg DM ha^−1^ on the plain in Spring 2025 (**Figure 5**). In contrast, unpalatable shrub biomass was lowest on the plain in Spring 2025 (1.94 kg DM ha^−1^) and highest in the valley in Autumn 2024 (38.27 kg DM ha^−1^). Unpalatable tree biomass remained low across all combinations, ranging from 0.33–0.42 kg DM ha^−1^ on the plain to 3.26 kg DM ha^−1^ in the valley in Autumn 2024. Consequently, total unpalatable biomass ranged from 5.68 kg DM ha^−1^ on the plain to 42.95 kg DM ha^−1^ in the valley during Autumn 2024.

Finally, estimated livestock carrying capacity ranged from 0.43 sheep units ha^−1^ per 90-day grazing period on the plain in Autumn 2024 to 0.77 sheep units ha^−1^ per 90-day grazing period in the valley in Spring 2024. Intermediate values of 0.67, 0.63, and 0.57 sheep units ha^−1^ per 90-day grazing period were recorded on the plain in Spring 2024, in the valley in Spring 2025, and on the plain in Spring 2025, respectively (**Figure 5**).

### 3.5 Principal component analysis

Principal component analysis (PCA) of the nine biomass and estimated livestock carrying-capacity variables showed that the first two principal components explained 59.5% of the total variance, with PC1 accounting for 35.5% and PC2 for 24.0%. PC1 was primarily represented associated with total palatable biomass (PC1 loading = 0.542), palatable tree biomass (PC1 loading = 0.493), and estimated livestock carrying capacity (PC1 loading = 0.451), all of which loaded positively on PC1 (**Figure 6**). In contrast, palatable herbaceous biomass and unpalatable herbaceous biomass were positioned along the negative PC1 axis.

**Figure 6:**
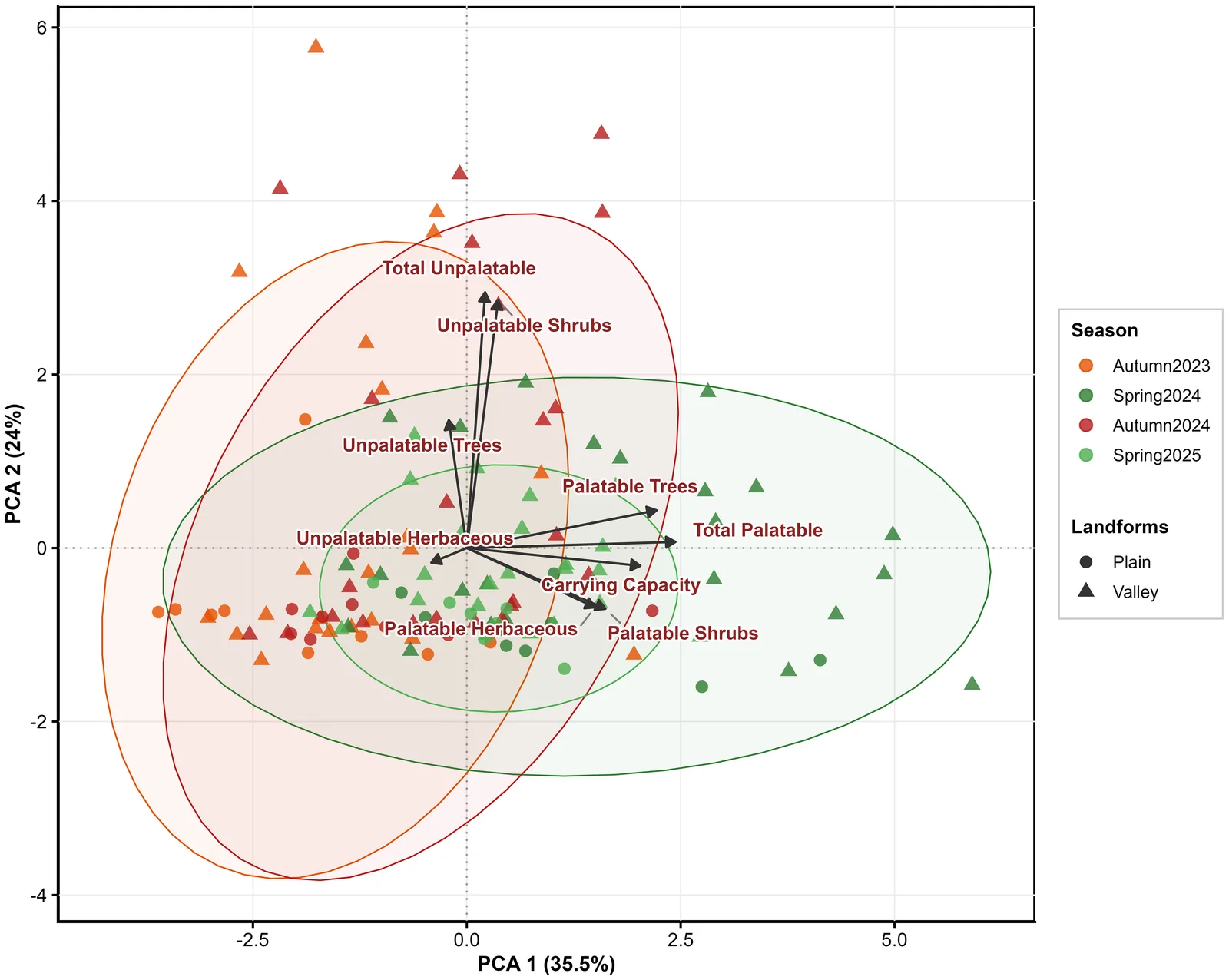
PCA biplot of biomass components and carrying capacity.

PC2, in turn, was associated primarily with unpalatable woody biomass. Total unpalatable biomass (PC2 loading = 0.655) and unpalatable shrub biomass (PC2 loading = 0.635) loaded strongly and positively on PC2, whereas the directions of palatable biomass components differed across variables. Moreover, estimated livestock carrying capacity had a negligible PC2 loading (−0.046).

Accordingly, observations from Spring 2024 and Spring 2025 generally occurred in the positive PC1 space, corresponding to higher values of total palatable biomass and estimated livestock carrying capacity. Conversely, autumn observations were more frequently distributed toward the negative PC1 space, although overlap among seasonal groups was evident. In addition, valley observations were more widely distributed toward positive PC2 values, particularly during the autumn campaigns (**Figure 6**).

## 4 Discussion

### 4.1 Seasonal dynamics of biomass and carrying capacity

The present study showed pronounced seasonal variation in palatable biomass at Bisha-1 rangeland, consistent with the water-limited functioning of arid ecosystems. Total palatable biomass was nearly 2.5-fold higher in Spring 2024 than in Autumn 2023 (**Figure 3D**). This seasonal pattern was reflected across all palatable components, although their responses differed in magnitude. For instance, herbaceous biomass showed the strongest fluctuations, while palatable shrub biomass showed a more moderate response. Meanwhile, palatable tree biomass remained the dominant and most stable component, contributing 70–80% of the total palatable biomass across all season–landform combinations. These findings indicate that seasonal changes in total forage availability were determined by both the rapid response of herbaceous vegetation and the persistent contribution of woody vegetation.

The stronger spring response of palatable biomass may be related to greater precipitation and its effects on soil-water availability, leaf development, photosynthetic activity, nutrient availability, and aboveground biomass accumulation (Luo et al., 2025). Herbaceous plants, particularly annual and short-lived perennial species, can respond rapidly to favorable moisture conditions but may senesce quickly as soil moisture declines. This result aligns with facts that precipitation drives forage production in water-limited grasslands while reduced rainfall limits ephemeral-plant survival, seed production, and biomass accumulation (Sala et al., 1988; Wang et al., 2022; He L et al., 2023a; Mu et al., 2023). Similarly, seasonal declines in herbaceous biomass and forage value have been reported in Mediterranean rangelands (Dickhoefer et al., 2010; Parlak et al., 2011; Alatürk et al., 2023), whereas higher winter–spring species richness and seed-bank density in the Rawdat Nourah ecosystem of Saudi Arabia further support the seasonal sensitivity of herbaceous vegetation in regional drylands (Al-Huqail et al., 2026). In contrast, the more moderate shrub response and the persistent dominance of palatable trees may reflect their perennial growth forms and efficient water-use strategies. This aligns with reports that woody vegetation can become dominant under grazing pressure and contribute to vegetation structural stability under environmental stress (Cheng et al., 2020; Yang et al., 2022; Biancari et al., 2024). These contrasting responses among biomass components are also consistent with resource-acquisition strategies in herbaceous plants and resource-conservation strategies in woody plants (Luo et al., 2026). Specifically, herbaceous plants employ resource-acquisition strategies that facilitate rapid biomass accumulation during periods of favorable moisture, whereas woody plants use resource-conservation strategies that maintain stable productivity responses to seasonal precipitation variability.

The climatic data provide context for the observed seasonal differences in palatable biomass. Thehighest total palatable biomass, recorded in Spring 2024, coincided with 96.57 mm ofprecipitation, whereas the lowest value, recorded in Autumn 2023, coincided with 16.74 mm of precipitation. In addition, spring precipitation varied markedly among the study years, from 127.40 mm in 2023 to 96.57 mm in 2024 and 46.08 mm in 2025. This interannual variability may alter soil-water availability and the timing and magnitude of seasonal plant growth, with consequent effects on palatable biomass. The 1990–2025 climatic record likewise shows substantial annual precipitation variability, with totals ranging from 59.78 to 180.20 mm (**Supplementary Table 5**). However, the three-year sequence alone is insufficient to establish a long-term drying trend.

Estimated livestock carrying capacity followed the seasonal pattern of palatable biomass, varying by approximately 1.5-fold among survey seasons and generally had higher values in spring than in autumn (**Figure 3I**). This seasonal pattern is consistent with reported variation in carrying capacity and with the non-equilibrium dynamics described for dryland grazing systems, in which forage availability responds strongly to variable moisture conditions (Vetter, 2005; López et al., 2022; Wang et al., 2024). Consequently, stocking levels should be adjusted according to seasonal forage availability rather than determined solely from annual averages. However, the present estimates represent dry-matter-based potential grazing capacity rather than direct animal intake. Future research should address this limitation by combining biomass estimates with direct measurements of seasonal forage nutritional quality (Ravhuhali et al., 2022; Alatürk et al., 2023).

### 4.2 Landform effects on vegetation productivity and forage availability

Landform affected total palatable biomass, which was 20.5% higher in valleys than on plains (**Figure 4D**). This landform-driven productivity differential aligns with foundational ecogeomorphic observations where valleys support substantially higher biomass compared to open plains (Dickhoefer et al., 2010). The higher biomass in valleys may be explained by their potential to retain soil moisture, concentrate runoff, and accumulate sediments and nutrients relative to open plains (Moeslund et al., 2013; Gxasheka and Dlamini, 2025). Furthermore, our findings reveal that spatial heterogeneity among biomass components is explicitly shaped by landform boundaries, reflecting how herbaceous and woody species sort themselves along highly localized resource gradients. This microtopographic sorting confirms that localized landform features act as primary environmental filters, governing plant community assembly, structural stratification, life-form distribution, and diversity patterns across arid landscapes (He X et al., 2023b; Zhang and Zhang, 2017; Lizarraga et al., 2025).

Specifically, the findings showed a clear landform difference in palatable tree biomass (**Figure 4C**), which may reflect hydrological advantages of low-lying terrain where run-off accumulation supports sustained woody vegetation growth. Indeed, local patterns in soil moisture may be influenced by topography through multiple mechanisms, including vertical distance to the groundwater level, drainage characteristics, and hydrological flow pathways (Moeslund et al., 2013). In contrast, the data indicated that palatable herbaceous and shrub biomass showed no statistically significant landform differences. This differential response suggests that herbaceous and shrub plants are less dependent on the accumulation of water and nutrients in valleys, which may primarily benefit deep-rooted woody species. This pattern aligns with historical observations that annual and short-lived perennial species exhibit more homogeneous spatial distributions compared to long-lived woody species, which may depend more strongly on sustained resource availability to maintain biomass (Noy-Meir, 1973; Aguiar and Sala, 1999; Reynolds et al., 2004).

Additionally, total unpalatable biomass components showed variable landform responses, with unpalatable shrub biomass more than sixfold higher in valleys than on plains (**Figure 4H**). This pattern reflects enhanced landscape heterogeneity in valley landforms, where valleys may act as areas of water and resource accumulation. Although unpalatable biomass does not directly contribute to livestock forage, it provides critical ecosystem services including soil stabilization, wildlife habitat, and carbon sequestration (Eldridge et al., 2011; Archer et al., 2017; Aweto, 2024). These findings suggest that valley landforms should be prioritized for conservation and restoration efforts, as their greater structural complexity and ecosystem service provision enhance overall rangeland resilience. Furthermore, the spatial concentration of unpalatable woody vegetation in valleys can buffer against soil erosion and reduce overgrazing pressure on palatable forage resources, thereby supporting sustainable rangeland management strategies that maintain both productivity and ecosystem health.

Despite the higher palatable biomass in valleys, estimated livestock carrying capacity did not differ significantly between landforms (**Figure 4I**). Given the unequal sampling allocation between plains (n = 9) and valleys (n = 23), this non-significant result should be interpreted cautiously and does not demonstrate equivalence between the two landforms. As carrying capacity was estimated from palatable dry matter rather than direct measurements of animal intake, future studies should incorporate direct assessments of forage quality, vegetation composition, physical forage accessibility, and livestock utilization to clarify the relationship between biomass and practical carrying capacity. This pattern aligns with observations from high-altitude grasslands, where localized overgrazing occurs in specific micro-habitats despite an overall regional surplus in carrying capacity (Saleem et al., 2024). Similarly, Zandler et al. (2023) demonstrated that high-biomass areas frequently exhibit low utilization rates due to accessibility restrictions imposed by rugged topography and distance from water, confirming that elevated primary productivity does not automatically translate to accessible forage.

### 4.3 Interactive effects of season and landform

The interaction between season and landform was non-significant for all biomass components and carrying capacity, indicating that seasonal variations remained consistent across landform types and that these factors operate independently. This structural consistency suggests that seasonal adjustments in stocking rates can be applied consistently across different landforms within the study area in terms of timing and magnitude. However, this finding does not imply that valleys and plains should be managed identically, as valleys concentrate woody vegetation, water resources, and dry-season refuge habitat that may require additional landform-specific safeguards beyond stocking rate adjustments. The additive nature of these drivers is illustrated by the absolute maximum and minimum values observed among factor combinations: spring 2024 in the valley yielded the highest total palatable biomass, whereas autumn 2023 on the plain recorded the lowest value, representing a 3.44-fold difference (**Figure 5D**). Within this additive framework, herbaceous biomass exhibited a notable numerical shift during Spring 2024, with plains recording higher localized yields than valleys (**Figure 5A**). This temporary increase suggests that under optimal seasonal moisture conditions, reduced competitive pressure from woody species on the plains can optimize short-term herbaceous growth, even within a landscape structurally dominated by valley productivity.

Despite this localized herbaceous advantage on the plains, valleys in Spring 2024 achieved the maximum carrying capacity, while plains in Autumn 2024 recorded the minimum (**Figure 5I**), representing a 1.75-fold difference. These findings demonstrate that while seasonal variation dominated carrying capacity patterns, landform effects remained a secondary but notable driver, confirming that carrying capacity depends on the integrated contribution of all palatable biomass components rather than herbaceous biomass alone. While some rangeland ecosystems show significant interactive effects between environmental drivers and management practices (Lasché et al., 2023; Smith et al., 2023), the results demonstrate that season and landform operate independently at Bisha-1.

Beyond the individual-variable results of the ANOVA, PCA provided an integrated view of the joint variation among biomass components and estimated livestock carrying capacity at Bisha-1 rangeland. Specifically, PC1 was associated primarily with total palatable biomass, palatable tree biomass, and estimated livestock carrying capacity, whereas PC2 captured variation in unpalatable woody biomass, with strong positive loadings for total unpalatable biomass and unpalatable shrubs (**Figure 6**). In addition, valley observations were more widely distributed toward positive PC2 values, corresponding to greater variation in unpalatable woody biomass. Together, the PCA results for Bisha-1 showed that higher total palatable biomass, palatable tree biomass, and estimated livestock carrying capacity covaried along PC1, whereas variation in unpalatable woody biomass was represented primarily along PC2, particularly among valley observations. This pattern is consistent with ecogeomorphic frameworks in which low-lying topographic features act as resource-convergence zones that foster vegetation structural heterogeneity in arid environments (Eldridge et al., 2011).

### 4.4 Management implications and adaptive strategies

The pronounced seasonal variation in palatable biomass and estimated livestock carrying capacity indicates that grazing capacity at Bisha-1 should be treated as a dynamic rather than a fixed attribute. Fixed stocking rates based on annual averages may expose the rangeland to excessive grazing pressure during low-forage periods and may underuse forage during more productive periods. Accordingly, grazing management should apply flexible stocking strategies that are adjusted seasonally according to current forage availability. Letsoalo et al. (2025) reported that grazing capacities in South African rangelands declined below national recommended norms during drought, illustrating the limitations of fixed stocking rates in variable dryland environments. Similarly, Derner and Augustine (2016) showed that setting stocking rates according to expected forage supply during dry or drought years can improve the resilience of rangeland plant–soil systems. These findings support the use of precautionary stocking decisions during low-rainfall periods at Bisha-1.

Landform variation should also be incorporated into adaptive grazing plans. Valleys contained greater woody biomass and higher unpalatable shrub biomass than plains, indicating differences in vegetation structure and potential ecosystem functions between the two landforms. Therefore, valleys may require landform-specific safeguards that balance livestock use with the protection of vegetation cover and sensitive soils. In contrast, plains should not be considered uniformly low-value grazing areas, because their forage availability also varied seasonally and they supported greater herbaceous biomass under some survey conditions.

Although the Season × Landform interaction was non-significant, indicating that seasonal patterns were broadly consistent between landforms, this result does not imply that valleys and plains should be managed identically. Seasonal adjustments in stocking timing can be applied across the study area, whereas the spatial distribution of grazing should account for differences in vegetation structure, forage condition, soil sensitivity, and local access. In practical terms, grazing pressure should be monitored across both valleys and plains, with protection of palatable herbaceous and shrub components during low-forage periods to allow adequate recovery. Such an approach combines seasonally responsive stocking with landform-aware management and may help maintain forage availability and rangeland condition over time.

### 4.5 Study limitations and future research

This study was conducted at a single rangeland site and included four survey campaigns over two years; consequently, the results may not capture the full range of longer-term climatic and vegetation variability or be directly generalizable to other Saudi Arabian rangelands. Estimated carrying capacity was based on palatable dry matter and a standardized grazing period, without direct measurements of forage nutritional quality or animal utilization. Seasonal changes in forage quality can affect the usable value of both herbaceous and woody forage. In addition, the climatic interpretation was based on TerraClimate and CHIRPS remote-sensing datasets rather than an on-site meteorological station. Future research should extend field monitoring across additional years and sites and combine biomass assessments with direct measurements of forage quality, accessibility, livestock use, and local climatic observations.

## 5 Conclusions

The findings of the present study reveal complex spatiotemporal dynamics in arid rangeland productivity that challenge conventional management approaches. The 1.5-fold seasonal variation in carrying capacity (0.72 sheep units ha^-1–^90 days^-1^ in spring 2024 to 0.48 sheep units ha^-1–^90 days^-1^ in autumn 2023) supports seasonality adjusted stocking strategies, whereas the non-significant difference in estimated carrying capacity across landforms (Valley: 0.61 sheep units ha^-1–^90 days^-1^; Plain: 0.53 sheep units ha^-1–^90 days^-1^) should be interpreted cautiously. These findings provide a practical basis for developing season-based grazing management strategies that address overgrazing challenges and support long-term ecological sustainability of arid rangelands in the Aseer region.

## Data Availability

The original contributions presented in the study are included in the article/**Supplementary Material**. Further inquiries can be directed to the corresponding author.
