# Supplementary material for: Seasonal and landform effects on plant productivity and carrying capacity at Bisha Rangeland, Saudi Arabia
Source: Front Plant Sci. 2026 Sep 18;17:1965543. doi: 10.3389/fpls.2026.1965543 (PMC13631829; doi:10.3389/fpls.2026.1965543)
Supplement: Supplementary file 1 [file Table1.docx]

Supplementary Material

# 1 Supplementary Data

**Supplementary Data 1.** Steps for normalized difference vegetation index (NDVI) calculation.

The Normalized Difference Vegetation Index (NDVI) was used to study both seasonal and annual changes in vegetation cover within the study sites. NDVI calculations relied on data derived from Sentinel-2 satellite imagery, developed by the European Space Agency (ESA). Sentinel-2 imagery contains 13 spectral bands in the visible, near-infrared (NIR), and short-wave infrared (SWIR) parts of the spectrum, with spatial resolutions of 10, 20, and 60 meters depending on the spectral bands. The field of view for Sentinel-2 satellite imagery is 290 km. NDVI was calculated using JavaScript programming language within the Google Earth Engine environment, following these steps:

1. Defining the study area (rangeland site).
2. Downloading satellite imagery covering the rangelands under study.
3. Constructing the mathematical equation for calculating the NDVI index.
4. Defining the spectral bands representing the terms of the NDVI equation (NIR and RED).
5. Applying the equation: NDVI = (NIR - RED) / (NIR + RED), where NIR is the spectral reflectance in the near-infrared range of spectrum, whereas RED is the spectral reflectance in the red range. For Sentinel-2, the NDVI was calculated according to the following equation: NDVI = (B8 - B4) / (B8 + B4).
6. Exporting the output results.
7. Processing and extraction of the vegetation cover was carried out using a threshold value according to (Holben, 1986). He found that bare-soil and non-vegetated areas exhibit NDVI values near 0.1 or lower, whereas active vegetation provides significantly higher values. A threshold value of 0.2 was adopted later as a conservative lower bound for vegetation detection.
8. Data post-processing and map production were carried out using ArcGIS-Pro (ver. 3.5) software.

# 2 Supplementary Figures and Tables

## 2.1 Supplementary Figures

Does not apply

**Supplementary Figure S1.** Photographs of livestock-owner interviews conducted during the three-day field-verification visits at the Bisha-1 rangeland site.

## 2.2 Supplementary Tables

**Supplementary Table 1.** Coordinates and landforms of sampling points in Bisha 1 rangeland site

| **Sampling point** | **Latitude** | **Longitude** | **Landform** |
| --- | --- | --- | --- |
| **1** | 19.519493 | 42.899180 | Valley |
| **2** | 19.510935 | 42.941730 | Valley |
| **3** | 19.551345 | 42.805587 | Valley |
| **4** | 19.554814 | 42.852071 | Valley |
| **5** | 19.557186 | 42.912327 | Valley |
| **6** | 19.560716 | 42.951060 | Plain |
| **7** | 19.610184 | 42.774316 | Valley |
| **8** | 19.596961 | 42.809913 | Plain |
| **9** | 19.598252 | 42.854062 | Plain |
| **10** | 19.607960 | 42.905171 | Valley |
| **11** | 19.607432 | 42.956102 | Valley |
| **12** | 19.635987 | 42.766775 | Plain |
| **13** | 19.636399 | 42.803465 | Valley |
| **14** | 19.644610 | 42.849660 | Valley |
| **15** | 19.634989 | 42.905366 | Valley |
| **16** | 19.654116 | 42.955226 | Valley |
| **17** | 19.683095 | 42.764494 | Valley |
| **18** | 19.680725 | 42.819743 | Valley |
| **19** | 19.694510 | 42.858967 | Valley |
| **20** | 19.692164 | 42.899362 | Valley |
| **21** | 19.694667 | 42.948718 | Valley |
| **22** | 19.746837 | 42.773313 | Valley |
| **23** | 19.739319 | 42.815927 | Valley |
| **24** | 19.732657 | 42.859945 | Plain |
| **25** | 19.742126 | 42.892178 | Plain |
| **26** | 19.722680 | 42.954196 | Valley |
| **27** | 19.793503 | 42.714534 | Plain |
| **28** | 19.816155 | 42.764420 | Valley |
| **29** | 19.773189 | 42.807187 | Valley |
| **30** | 19.783313 | 42.846625 | Plain |
| **31** | 19.776743 | 42.896395 | Plain |
| **32** | 19.802046 | 42.940989 | Valley |

Supplementary Table 2. Scientific name, family, life form, lifespan and palatability of recorded plants at Bisha-1 site.

| **No.** | **Scientific name** | **Family** | **Life form** | **lifespan** | **Palatability** |
| --- | --- | --- | --- | --- | --- |
| 1. | *Blepharis ciliaris* | Acanthaceae | Herb | Perennial | Palatable |
| 2. | *Aizoon canarinse* | Aizoaceae | Herb | Annual | Palatable |
| 3. | *Aerva javanica* | Amaranthaceae | Shrub | Perennial | Palatable |
| 4. | *Calotropis procera* | Apocynaceae | Tree | Perennial | Unpalatable |
| 5. | *Leptadenia pyrotechnica* | Apocynaceae | Shrub | Perennial | Palatable |
| 6. | *Odontanthera radians* | Apocynaceae | Herb | Annual | Unpalatable |
| 7. | *Pergularia tomentosa* | Apocynaceae | Shrub | Perennial | Unpalatable |
| 8. | *Rhazya stricta* | Apocynaceae | Shrub | Perennial | Unpalatable |
| 9. | *Xanthium strumarium* | Asteraceae | Herb | Perennial | Unpalatable |
| 10. | *Arnebia decumbens* | Boraginaceae | Herb | Annual | Palatable |
| 11. | *Heliotropium crispum* | Boraginaceae | Shrub | Perennial | Unpalatable |
| 12. | *Farsetia heliophila* | Brassicaceae | Herb | Perennial | Palatable |
| 13. | *Morettia parviflora* | Brassicaceae | Herb | Annual | Palatable |
| 14. | *Commiphora kataf* | Burseraceae | Tree | Perennial | Palatable |
| 15. | *Commiphora myrrah* | Burseraceae | Tree | Perennial | Palatable |
| 16. | *Commiphora gileadensis* | Burseraceae | Tree | Perennial | Palatable |
| 17. | *Cleome pallida* | Capparaceae | Herb | Perennial | Palatable |
| 18. | *Maerua crassifolia* | Capparaceae | Tree | Perennial | Palatable |
| 19. | *Gymnocarpos sclerocephalus* | Caryophyllaceae | Herb | Annual | Unpalatable |
| 20. | *Polycarpeae repens* | Caryophyllaceae | Herb | Annual | Palatable |
| 21. | *Cleome gaucescens* | Cleomaceae | Herb | Annual | Palatable |
| 22. | *Convolvulus buschiricus* | Convolvolaceae | Herb | Annual | Palatable |
| 23. | *Citrullus colocynthis* | Cucurbitaceae | Herb | Perennial | Unpalatable |
| 24. | *Cucumis prophetraum* | Cucurbitaceae | Herb | Annual | Unpalatable |
| 25. | *Chrozophora tinctoria* | Euphorbiaceae | Shrub | Perennial | Unpalatable |
| 26. | *Astragalus dactylocapus* | Fabaceae | Herb | Perennial | Palatable |
| 27. | *Astraglus sparsus* | Fabaceae | Herb | Annual | Palatable |
| 28. | *Crottalaria emarginella* | Fabaceae | Herb | Annual | Unpalatable |
| 29. | *Crottalaria leptocarpa* | Fabaceae | Herb | Annual | Palatable |
| 30. | *Delonix elata* | Fabaceae | Tree | Perennial | Palatable |
| 31. | *Indigofera spinosa* | Fabaceae | Shrub | Perennial | Palatable |
| 32. | *Senegalia asak* | Fabaceae | Tree | Perennial | Palatable |
| 33. | *Senegalia hamulosa* | Fabaceae | Tree | Perennial | Palatable |
| 34. | *Senna italica* | Fabaceae | Shrub | Perennial | Unpalatable |
| 35. | *Tephorsia nubica* | Fabaceae | Shrub | Perennial | Unpalatable |
| 36. | *Vachellia flava* | Fabaceae | Tree | Perennial | Palatable |
| 37. | *Vachellia oerfota* | Fabaceae | Tree | Perennial | Palatable |
| 38. | *Vachellia tortilis* | Fabaceae | Tree | Perennial | Palatable |
| 39. | *Monsonia nivea* | Geraniaceae | Herb | Perennial | Palatable |
| 40. | *Gisekia pharnaceoides* | Gisekiaceae | Herb | Annual | Palatable |
| 41. | *Leucas inflata* | Lamiaceae | Herb | Annual | Unpalatable |
| 42. | *Limeum obovatum* | Lamiaceae | Herb | Annual | Palatable |
| 43. | *Neurada procumbens* | Neuradaceae | Herb | Annual | Palatable |
| 44. | *Boerhavia coccineae* | Nyctaginaceae | Herb | Annual | Palatable |
| 45. | *Olea europaea* | Oleaceae | Tree | Perennial | Palatable |
| 46. | *Eragrostis barrelieri* | Poaceae | Herb | Annual | Palatable |
| 47. | *Hordeum murinum* | Poaceae | Herb | Annual | Palatable |
| 48. | *Hyparrhenia hirta* | Poaceae | Herb | Perennial | Palatable |
| 49. | *Lasiurus scindicus* | Poaceae | Herb | Perennial | Palatable |
| 50. | *Panicum turgidum* | Poaceae | Herb | Perennial | Palatable |
| 51. | *Stipagrostis hiritigluma* | Poaceae | Herb | Annual | Palatable |
| 52. | *Stipagrostis plumosa* | Poaceae | Herb | Perennial | Palatable |
| 53. | *Cymbopogon commutatus* | Poaceae | Herb | Perennial | Palatable |
| 54. | *Pennosetum divisum* | Poaceae | Herb | Perennial | Palatable |
| 55. | *Stipagrostis cilata* | Poaceae | Herb | Perennial | Palatable |
| 56. | *Ziziphus nummularia* | Rhamnaceae | Tree | Perennial | Palatable |
| 57. | *Salvadora persica* | Salvadoraceae | Tree | Perennial | Palatable |
| 58. | *Lycium shawii* | Solanaceae | Tree | Perennial | Palatable |
| 59. | *Seetzenia lanata* | Zygophyllaceae | Herb | Annual | Palatable |
| 60. | *Tribulus pentandrus* | Zygophyllaceae | Herb | Annual | Palatable |
| 61. | *Zygophyllum bruguieri* | Zygophyllaceae | Herb | Perennial | Palatable |

**Supplementary Table 3**. Seasonal and annual maximum temperature of Bisha area from 1990 to 2025.

| Season | Winter | | | Spring | | | Summer | | | Fall | | | Annual | | |
| --- | --- | --- | --- | --- | --- | --- | --- | --- | --- | --- | --- | --- | --- | --- | --- |
| Year | Max. | Min. | Avg. | Max. | Min. | Avg. | Max. | Min. | Avg. | Max. | Min. | Avg. | Max. | Min. | Avg. |
| 1990 | 29.05 | 25.20 | 27.02 | 37.66 | 29.02 | 33.24 | 39.77 | 37.11 | 38.45 | 38.03 | 31.16 | 34.59 | 39.77 | 25.20 | 33.32 |
| 1991 | 27.91 | 24.96 | 26.26 | 38.56 | 26.97 | 33.53 | 39.69 | 37.11 | 38.51 | 37.82 | 29.07 | 33.66 | 39.69 | 24.96 | 32.99 |
| 1992 | 27.89 | 24.51 | 25.82 | 36.70 | 29.11 | 33.21 | 38.88 | 35.76 | 37.55 | 37.20 | 29.12 | 33.14 | 38.88 | 24.51 | 32.43 |
| 1993 | 27.41 | 25.53 | 26.68 | 36.39 | 31.00 | 32.99 | 39.29 | 37.84 | 38.79 | 38.40 | 30.80 | 34.68 | 39.29 | 25.53 | 33.29 |
| 1994 | 28.59 | 26.78 | 27.61 | 38.20 | 29.81 | 34.14 | 39.52 | 37.88 | 38.59 | 38.46 | 32.58 | 35.40 | 39.52 | 26.78 | 33.94 |
| 1995 | 28.58 | 26.62 | 27.56 | 37.19 | 29.98 | 33.45 | 40.18 | 38.12 | 38.85 | 38.36 | 30.57 | 34.59 | 40.18 | 26.62 | 33.61 |
| 1996 | 29.21 | 26.17 | 27.67 | 37.03 | 30.31 | 33.50 | 39.45 | 37.93 | 38.49 | 40.03 | 29.99 | 35.10 | 40.03 | 26.17 | 33.69 |
| 1997 | 27.70 | 26.71 | 27.10 | 37.03 | 29.40 | 33.44 | 40.62 | 38.14 | 39.64 | 38.75 | 30.96 | 35.04 | 40.62 | 26.71 | 33.81 |
| 1998 | 29.44 | 26.15 | 28.07 | 38.55 | 30.51 | 34.41 | 41.19 | 39.08 | 40.34 | 38.50 | 31.19 | 34.71 | 41.19 | 26.15 | 34.38 |
| 1999 | 30.96 | 27.35 | 28.97 | 39.45 | 30.34 | 34.92 | 40.51 | 37.91 | 39.39 | 39.40 | 31.05 | 35.03 | 40.51 | 27.35 | 34.58 |
| 2000 | 28.58 | 26.27 | 27.41 | 38.15 | 31.06 | 34.72 | 40.36 | 39.45 | 39.87 | 39.13 | 30.01 | 34.89 | 40.36 | 26.27 | 34.22 |
| 2001 | 28.94 | 26.10 | 27.81 | 38.43 | 32.87 | 35.26 | 39.51 | 38.61 | 39.17 | 39.42 | 30.79 | 35.09 | 39.51 | 26.10 | 34.33 |
| 2002 | 28.92 | 26.64 | 28.02 | 38.91 | 30.88 | 34.51 | 41.02 | 38.73 | 39.85 | 39.39 | 31.19 | 35.29 | 41.02 | 26.64 | 34.42 |
| 2003 | 29.78 | 26.60 | 28.07 | 38.15 | 32.69 | 35.17 | 40.30 | 38.22 | 39.44 | 38.85 | 31.05 | 35.09 | 40.30 | 26.60 | 34.44 |
| 2004 | 29.34 | 26.18 | 27.90 | 38.56 | 32.36 | 35.07 | 40.52 | 37.70 | 39.47 | 38.13 | 31.07 | 34.68 | 40.52 | 26.18 | 34.28 |
| 2005 | 29.83 | 24.69 | 27.46 | 35.54 | 31.09 | 33.55 | 40.18 | 37.99 | 39.20 | 38.62 | 30.19 | 34.82 | 40.18 | 24.69 | 33.76 |
| 2006 | 29.61 | 25.76 | 27.54 | 37.09 | 32.08 | 34.27 | 40.31 | 38.90 | 39.81 | 38.73 | 31.84 | 35.06 | 40.31 | 25.76 | 34.17 |
| 2007 | 29.40 | 24.89 | 27.40 | 38.41 | 31.60 | 34.87 | 40.27 | 38.11 | 38.99 | 39.00 | 31.49 | 35.12 | 40.27 | 24.89 | 34.10 |
| 2008 | 27.79 | 25.37 | 26.53 | 38.33 | 33.24 | 35.59 | 40.53 | 37.99 | 38.86 | 39.30 | 30.84 | 34.94 | 40.53 | 25.37 | 33.98 |
| 2009 | 30.49 | 26.23 | 28.56 | 39.16 | 30.92 | 35.11 | 41.41 | 38.16 | 39.99 | 37.99 | 32.24 | 34.67 | 41.41 | 26.23 | 34.58 |
| 2010 | 29.85 | 26.02 | 27.48 | 38.83 | 32.11 | 35.15 | 41.40 | 39.59 | 40.47 | 39.29 | 29.66 | 34.94 | 41.40 | 26.02 | 34.51 |
| 2011 | 30.08 | 26.33 | 27.63 | 38.28 | 29.88 | 34.58 | 41.01 | 39.42 | 39.98 | 39.65 | 31.21 | 35.49 | 41.01 | 26.33 | 34.42 |
| 2012 | 28.84 | 27.59 | 28.40 | 38.93 | 30.76 | 34.22 | 40.98 | 38.57 | 39.91 | 38.62 | 31.49 | 35.18 | 40.98 | 27.59 | 34.43 |
| 2013 | 30.67 | 27.14 | 28.39 | 38.07 | 32.09 | 34.79 | 40.89 | 37.81 | 39.10 | 37.53 | 31.04 | 34.64 | 40.89 | 27.14 | 34.23 |
| 2014 | 34.23 | 27.63 | 30.18 | 38.06 | 32.17 | 35.11 | 40.60 | 38.24 | 39.59 | 37.89 | 30.17 | 34.25 | 40.60 | 27.63 | 34.79 |
| 2015 | 30.29 | 26.37 | 27.84 | 37.52 | 32.11 | 35.16 | 40.36 | 38.90 | 39.75 | 39.56 | 32.07 | 35.95 | 40.36 | 26.37 | 34.67 |
| 2016 | 28.39 | 24.75 | 27.15 | 35.92 | 32.72 | 33.85 | 40.08 | 38.48 | 39.32 | 38.65 | 30.11 | 34.43 | 40.08 | 24.75 | 33.69 |
| 2017 | 27.84 | 25.60 | 26.41 | 36.87 | 30.81 | 34.46 | 40.70 | 38.46 | 39.92 | 38.82 | 30.33 | 34.63 | 40.70 | 25.60 | 33.86 |
| 2018 | 30.51 | 26.06 | 28.16 | 37.81 | 33.19 | 35.05 | 40.20 | 38.18 | 39.41 | 39.39 | 30.44 | 34.96 | 40.20 | 26.06 | 34.39 |
| 2019 | 30.25 | 28.97 | 29.58 | 36.75 | 31.20 | 34.39 | 41.70 | 38.59 | 40.53 | 40.41 | 32.05 | 36.39 | 41.70 | 28.97 | 35.22 |
| 2020 | 29.79 | 25.93 | 28.08 | 37.79 | 31.43 | 34.78 | 40.71 | 38.02 | 39.58 | 38.91 | 31.45 | 34.86 | 40.71 | 25.93 | 34.33 |
| 2021 | 28.56 | 26.29 | 27.56 | 38.58 | 33.08 | 35.26 | 40.77 | 38.80 | 39.86 | 39.40 | 31.56 | 35.69 | 40.77 | 26.29 | 34.59 |
| 2022 | 28.99 | 27.93 | 28.29 | 38.43 | 31.90 | 35.21 | 41.02 | 37.55 | 39.52 | 39.30 | 32.15 | 35.49 | 41.02 | 27.93 | 34.63 |
| 2023 | 28.93 | 27.60 | 28.31 | 34.16 | 32.6 | 33.46 | 41.33 | 39.33 | 40.5 | 37.29 | 35.57 | 36.51 | 35.41 | 33.77 | 34.7 |
| 2024 | 29.60 | 28.23 | 28.99 | 35.45 | 33.93 | 34.77 | 41.1 | 39.10 | 40.28 | 37.51 | 35.77 | 36.74 | 35.90 | 34.28 | 35.21 |
| 2025 | 28.86 | 27.30 | 28.14 | 40.23 | 33.96 | 37.36 | 41.23 | 40.35 | 40.67 | 38.37 | 30.74 | 35.20 | 41.23 | 27.30 | 35.34 |

**Supplementary Table 4**. Seasonal and annual minimum temperature of Bisha area from 1990 to 2025.

| Season | Winter | | | Spring | | | Summer | | | Fall | | | Annual | | |
| --- | --- | --- | --- | --- | --- | --- | --- | --- | --- | --- | --- | --- | --- | --- | --- |
| Year | Max. | Min. | Avg. | Max. | Min. | Avg. | Max. | Min. | Avg. | Max. | Min. | Avg. | Max. | Min. | Avg. |
| 1990 | 13.03 | 10.96 | 11.84 | 21.93 | 15.04 | 18.42 | 26.67 | 23.46 | 25.14 | 22.85 | 14.76 | 18.67 | 26.67 | 10.96 | 18.52 |
| 1991 | 13.58 | 11.68 | 12.51 | 23.60 | 17.85 | 20.40 | 25.85 | 23.81 | 24.77 | 21.55 | 12.79 | 17.27 | 25.85 | 11.68 | 18.74 |
| 1992 | 12.30 | 9.26 | 10.43 | 21.76 | 14.34 | 18.20 | 24.50 | 23.01 | 23.80 | 20.93 | 12.83 | 16.75 | 24.50 | 9.26 | 17.29 |
| 1993 | 11.81 | 10.24 | 11.16 | 21.47 | 16.22 | 17.98 | 26.58 | 23.41 | 25.04 | 22.13 | 14.51 | 18.29 | 26.58 | 10.24 | 18.12 |
| 1994 | 12.88 | 11.49 | 12.08 | 23.25 | 15.04 | 19.13 | 26.62 | 23.64 | 24.84 | 22.18 | 16.29 | 19.01 | 26.62 | 11.49 | 18.76 |
| 1995 | 12.87 | 11.34 | 12.03 | 22.24 | 15.21 | 18.43 | 26.86 | 24.14 | 25.09 | 22.09 | 14.28 | 18.20 | 26.86 | 11.34 | 18.44 |
| 1996 | 13.50 | 10.89 | 12.19 | 22.24 | 15.74 | 18.72 | 26.99 | 24.24 | 25.59 | 21.67 | 13.19 | 17.23 | 26.99 | 10.89 | 18.43 |
| 1997 | 11.79 | 10.57 | 11.01 | 21.60 | 13.10 | 17.13 | 25.39 | 23.64 | 24.44 | 22.79 | 13.67 | 17.84 | 25.39 | 10.57 | 17.60 |
| 1998 | 13.15 | 10.86 | 12.21 | 22.52 | 14.40 | 18.70 | 26.93 | 23.96 | 25.26 | 22.52 | 15.21 | 18.59 | 26.93 | 10.86 | 18.69 |
| 1999 | 16.26 | 10.83 | 13.12 | 22.02 | 15.77 | 18.76 | 26.96 | 23.48 | 24.98 | 21.64 | 15.08 | 18.31 | 26.96 | 10.83 | 18.79 |
| 2000 | 12.87 | 10.64 | 11.59 | 23.36 | 14.26 | 19.19 | 26.90 | 24.12 | 25.83 | 20.97 | 13.22 | 17.70 | 26.90 | 10.64 | 18.58 |
| 2001 | 13.54 | 9.56 | 11.53 | 23.49 | 15.98 | 19.66 | 26.86 | 23.68 | 25.38 | 21.96 | 13.90 | 17.79 | 26.86 | 9.56 | 18.59 |
| 2002 | 13.24 | 11.35 | 12.50 | 23.97 | 16.11 | 19.50 | 27.18 | 24.10 | 26.07 | 21.73 | 14.29 | 18.30 | 27.18 | 11.35 | 19.09 |
| 2003 | 13.08 | 11.59 | 12.34 | 23.12 | 16.29 | 19.39 | 27.27 | 23.97 | 25.24 | 21.99 | 15.08 | 18.29 | 27.27 | 11.59 | 18.82 |
| 2004 | 12.35 | 11.65 | 11.89 | 22.12 | 16.46 | 18.88 | 27.05 | 22.27 | 24.83 | 22.05 | 15.08 | 18.44 | 27.05 | 11.65 | 18.51 |
| 2005 | 14.14 | 10.26 | 12.75 | 23.41 | 15.78 | 19.80 | 27.54 | 23.95 | 25.59 | 22.35 | 14.39 | 18.01 | 27.54 | 10.26 | 19.04 |
| 2006 | 13.58 | 10.31 | 12.10 | 23.36 | 15.07 | 18.89 | 26.75 | 24.61 | 25.54 | 21.46 | 15.56 | 18.56 | 26.75 | 10.31 | 18.77 |
| 2007 | 12.50 | 9.66 | 11.52 | 23.27 | 15.30 | 19.39 | 26.56 | 23.85 | 25.01 | 22.14 | 15.19 | 18.45 | 26.56 | 9.66 | 18.59 |
| 2008 | 12.46 | 10.35 | 11.21 | 21.59 | 16.63 | 19.41 | 27.05 | 23.40 | 25.66 | 23.34 | 13.86 | 18.21 | 27.05 | 10.35 | 18.62 |
| 2009 | 14.79 | 10.94 | 13.03 | 24.22 | 15.32 | 19.56 | 27.31 | 25.53 | 26.37 | 24.23 | 15.95 | 20.00 | 27.31 | 10.94 | 19.74 |
| 2010 | 14.16 | 10.42 | 11.96 | 22.21 | 17.33 | 19.68 | 28.32 | 25.54 | 26.72 | 23.01 | 13.38 | 18.54 | 28.32 | 10.42 | 19.23 |
| 2011 | 14.39 | 10.88 | 12.10 | 23.35 | 15.09 | 19.57 | 26.88 | 23.97 | 25.74 | 23.38 | 14.94 | 19.11 | 26.88 | 10.88 | 19.13 |
| 2012 | 13.18 | 12.30 | 12.87 | 24.00 | 15.98 | 19.22 | 27.63 | 24.30 | 26.27 | 22.67 | 14.80 | 18.79 | 27.63 | 12.30 | 19.29 |
| 2013 | 14.64 | 11.55 | 12.75 | 22.64 | 16.91 | 19.48 | 26.77 | 25.55 | 26.13 | 23.47 | 14.85 | 19.02 | 26.77 | 11.55 | 19.34 |
| 2014 | 18.94 | 12.05 | 14.78 | 21.83 | 16.87 | 19.47 | 27.30 | 23.78 | 25.49 | 23.03 | 14.68 | 18.66 | 27.30 | 12.05 | 19.60 |
| 2015 | 14.78 | 11.04 | 12.41 | 23.97 | 18.30 | 20.94 | 27.64 | 24.36 | 26.15 | 23.60 | 14.87 | 19.04 | 27.64 | 11.04 | 19.64 |
| 2016 | 12.84 | 11.07 | 12.19 | 21.70 | 18.17 | 19.37 | 27.99 | 23.64 | 26.40 | 22.69 | 13.92 | 18.20 | 27.99 | 11.07 | 19.04 |
| 2017 | 12.56 | 8.91 | 11.30 | 22.14 | 16.11 | 19.36 | 27.56 | 24.59 | 26.48 | 22.81 | 15.17 | 18.67 | 27.56 | 8.91 | 18.95 |
| 2018 | 14.63 | 10.63 | 12.45 | 23.28 | 18.07 | 19.95 | 27.59 | 24.29 | 26.01 | 23.35 | 14.08 | 18.59 | 27.59 | 10.63 | 19.25 |
| 2019 | 14.56 | 13.68 | 14.05 | 21.81 | 16.42 | 19.38 | 27.59 | 25.43 | 26.78 | 24.13 | 15.78 | 20.00 | 27.59 | 13.68 | 20.05 |
| 2020 | 14.11 | 10.77 | 12.59 | 22.74 | 16.45 | 19.70 | 26.73 | 24.73 | 25.70 | 22.58 | 15.15 | 18.46 | 26.73 | 10.77 | 19.11 |
| 2021 | 12.89 | 11.13 | 12.06 | 23.54 | 18.09 | 20.18 | 27.52 | 24.79 | 25.99 | 23.07 | 15.25 | 19.29 | 27.52 | 11.13 | 19.38 |
| 2022 | 13.31 | 12.35 | 12.79 | 23.34 | 16.76 | 20.01 | 26.20 | 25.00 | 25.68 | 22.97 | 15.82 | 19.01 | 26.20 | 12.35 | 19.37 |
| 2023 | 13.3 | 12.23 | 12.8 | 18.84 | 17.5 | 18.25 | 27.6 | 25.4 | 26.67 | 20.79 | 19.17 | 20.03 | 20.11 | 18.57 | 19.43 |
| 2024 | 13.97 | 12.87 | 13.48 | 20.12 | 18.83 | 19.56 | 27.34 | 25.17 | 26.44 | 21.01 | 19.37 | 20.25 | 20.59 | 19.08 | 19.95 |
| 2025 | 13.2 | 11.8 | 12.56 | 20.44 | 12.55 | 16.82 | 21.67 | 20.42 | 21.16 | 19.99 | 8.01 | 13.79 | 21.67 | 8.01 | 16.08 |

**Supplementary Table 5**. Seasonal and annual precipitation of Bisha area from 1990 to 2025.

| Season | Winter | | | Spring | | | Summer | | | Fall | | | Annual | | | |
| --- | --- | --- | --- | --- | --- | --- | --- | --- | --- | --- | --- | --- | --- | --- | --- | --- |
| Year | Max. | Min. | Avg. | Max. | Min. | Avg. | Max. | Min. | Avg. | Max. | Min. | Avg. | Max. | Min. | Avg. | Total |
| 1990 | 1.03 | 0.00 | 0.34 | 81.05 | 3.88 | 34.81 | 7.55 | 0.00 | 2.52 | 0.00 | 0.00 | 0.00 | 81.05 | 0.00 | 9.42 | 113.02 |
| 1991 | 5.16 | 0.00 | 2.06 | 46.30 | 14.88 | 27.58 | 8.94 | 0.00 | 3.11 | 3.11 | 0.00 | 1.04 | 46.30 | 0.00 | 8.45 | 101.36 |
| 1992 | 8.74 | 4.44 | 7.13 | 18.23 | 5.25 | 10.21 | 30.02 | 0.00 | 10.40 | 18.53 | 0.00 | 6.75 | 30.02 | 0.00 | 8.62 | 103.47 |
| 1993 | 4.90 | 0.00 | 2.88 | 31.82 | 17.70 | 24.18 | 9.86 | 0.00 | 3.29 | 6.24 | 0.00 | 3.63 | 31.82 | 0.00 | 8.50 | 101.94 |
| 1994 | 1.27 | 0.00 | 0.42 | 28.47 | 13.37 | 19.44 | 8.81 | 0.00 | 2.94 | 2.79 | 0.00 | 1.02 | 28.47 | 0.00 | 5.96 | 71.47 |
| 1995 | 7.58 | 2.77 | 4.94 | 32.86 | 10.20 | 25.31 | 9.77 | 2.06 | 4.73 | 4.56 | 0.00 | 1.61 | 32.86 | 0.00 | 9.15 | 109.77 |
| 1996 | 5.15 | 1.01 | 2.93 | 26.57 | 14.85 | 21.09 | 7.54 | 0.01 | 2.96 | 13.04 | 0.00 | 4.35 | 26.57 | 0.00 | 7.83 | 93.98 |
| 1997 | 2.71 | 0.00 | 1.31 | 23.60 | 7.74 | 16.61 | 6.80 | 0.00 | 3.48 | 14.47 | 0.00 | 8.33 | 23.60 | 0.00 | 7.43 | 89.20 |
| 1998 | 8.16 | 0.00 | 3.25 | 48.98 | 28.97 | 39.43 | 8.58 | 0.00 | 3.50 | 0.04 | 0.00 | 0.01 | 48.98 | 0.00 | 11.55 | 138.59 |
| 1999 | 13.29 | 0.00 | 5.83 | 26.66 | 0.19 | 12.58 | 7.19 | 0.00 | 2.60 | 1.40 | 0.00 | 0.89 | 26.66 | 0.00 | 5.47 | 65.68 |
| 2000 | 4.61 | 0.00 | 2.00 | 47.28 | 5.42 | 24.64 | 1.64 | 0.00 | 0.55 | 13.88 | 0.23 | 5.30 | 47.28 | 0.00 | 8.12 | 97.47 |
| 2001 | 4.26 | 0.51 | 2.34 | 52.60 | 0.41 | 21.56 | 5.08 | 0.00 | 1.74 | 3.98 | 0.00 | 1.33 | 52.60 | 0.00 | 6.74 | 80.90 |
| 2002 | 2.51 | 0.00 | 1.14 | 29.29 | 4.10 | 17.30 | 1.43 | 0.00 | 0.48 | 4.22 | 0.00 | 2.32 | 29.29 | 0.00 | 5.31 | 63.72 |
| 2003 | 3.32 | 0.00 | 2.02 | 22.48 | 5.36 | 13.71 | 9.81 | 0.00 | 3.60 | 2.58 | 0.00 | 1.31 | 22.48 | 0.00 | 5.16 | 61.93 |
| 2004 | 4.84 | 0.00 | 3.18 | 132.33 | 0.00 | 51.20 | 7.09 | 0.00 | 2.36 | 2.54 | 0.00 | 1.23 | 132.33 | 0.00 | 14.49 | 173.92 |
| 2005 | 4.38 | 0.10 | 1.68 | 67.02 | 7.56 | 31.17 | 2.54 | 0.00 | 1.20 | 3.43 | 0.00 | 1.14 | 67.02 | 0.00 | 8.80 | 105.61 |
| 2006 | 2.61 | 1.55 | 2.02 | 102.36 | 11.48 | 47.14 | 1.52 | 0.00 | 0.51 | 3.30 | 0.00 | 1.19 | 102.36 | 0.00 | 12.71 | 152.58 |
| 2007 | 1.59 | 0.50 | 1.00 | 46.21 | 4.69 | 23.56 | 5.47 | 0.00 | 1.83 | 0.00 | 0.00 | 0.00 | 46.21 | 0.00 | 6.60 | 79.17 |
| 2008 | 4.30 | 0.00 | 1.43 | 30.33 | 13.24 | 19.79 | 1.43 | 0.00 | 0.48 | 9.85 | 0.00 | 3.74 | 30.33 | 0.00 | 6.36 | 76.34 |
| 2009 | 6.48 | 1.02 | 4.04 | 24.26 | 5.47 | 13.05 | 1.78 | 0.80 | 1.31 | 2.67 | 0.00 | 1.53 | 24.26 | 0.00 | 4.98 | 59.78 |
| 2010 | 3.61 | 0.00 | 1.98 | 57.63 | 12.36 | 29.06 | 8.02 | 0.00 | 4.67 | 5.43 | 0.00 | 2.61 | 57.63 | 0.00 | 9.58 | 114.92 |
| 2011 | 0.01 | 0.00 | 0.00 | 36.07 | 9.39 | 24.17 | 8.39 | 0.00 | 2.80 | 1.65 | 0.00 | 0.57 | 36.07 | 0.00 | 6.89 | 82.63 |
| 2012 | 2.51 | 0.00 | 0.84 | 60.61 | 7.22 | 29.16 | 5.97 | 0.00 | 1.99 | 4.81 | 0.00 | 2.62 | 60.61 | 0.00 | 8.65 | 103.82 |
| 2013 | 0.94 | 0.00 | 0.53 | 34.44 | 14.92 | 24.11 | 10.75 | 0.72 | 5.20 | 12.56 | 0.00 | 4.50 | 34.44 | 0.00 | 8.59 | 103.04 |
| 2014 | 0.00 | 0.00 | 0.00 | 29.78 | 13.05 | 20.52 | 6.51 | 0.00 | 2.17 | 5.68 | 0.02 | 2.54 | 29.78 | 0.00 | 6.31 | 75.70 |
| 2015 | 6.38 | 2.29 | 3.84 | 27.12 | 5.73 | 15.86 | 8.88 | 0.00 | 3.12 | 2.90 | 0.00 | 1.04 | 27.12 | 0.00 | 5.97 | 71.61 |
| 2016 | 0.25 | 0.00 | 0.09 | 105.67 | 15.43 | 46.40 | 14.46 | 1.89 | 8.53 | 14.92 | 0.00 | 5.05 | 105.67 | 0.00 | 15.02 | 180.20 |
| 2017 | 11.84 | 0.00 | 3.96 | 29.45 | 11.99 | 21.61 | 5.13 | 0.00 | 1.71 | 2.62 | 0.00 | 0.91 | 29.45 | 0.00 | 7.05 | 84.56 |
| 2018 | 2.14 | 0.00 | 0.77 | 67.61 | 16.54 | 35.40 | 6.57 | 0.00 | 2.35 | 13.58 | 0.00 | 7.78 | 67.61 | 0.00 | 11.57 | 138.89 |
| 2019 | 6.52 | 1.37 | 3.16 | 18.66 | 15.18 | 16.64 | 10.99 | 0.00 | 4.40 | 7.06 | 0.00 | 2.76 | 18.66 | 0.00 | 6.74 | 80.87 |
| 2020 | 2.72 | 0.00 | 0.91 | 19.17 | 17.44 | 18.57 | 17.88 | 1.64 | 8.83 | 10.05 | 0.00 | 3.98 | 19.17 | 0.00 | 8.07 | 96.84 |
| 2021 | 3.72 | 0.38 | 1.54 | 29.51 | 10.10 | 17.79 | 11.89 | 0.00 | 6.36 | 0.62 | 0.00 | 0.31 | 29.51 | 0.00 | 6.50 | 78.03 |
| 2022 | 4.82 | 0.00 | 2.22 | 20.32 | 0.00 | 11.35 | 26.68 | 1.05 | 10.60 | 1.57 | 0.00 | 0.62 | 26.68 | 0.00 | 6.20 | 74.40 |
| 2023 | 13.39 | 10.11 | 11.58 | 143.92 | 116.87 | 127.4 | 21.26 | 10.57 | 15.4 | 20.37 | 13.73 | 16.74 | 143.92 | 10.11 | 42.78 | 165.64 |
| 2024 | 17.97 | 9.62 | 12.81 | 119.04 | 82.96 | 96.57 | 45.18 | 13.27 | 26.48 | 21.33 | 15.23 | 17.91 | 119.04 | 9.62 | 38.44 | 150.27 |
| 2025 | 12.62 | 8.18 | 10.00 | 48.95 | 42.42 | 46.08 | 31.22 | 0.91 | 11.03 | 4.13 | 0.02 | 1.89 | 48.95 | 0.02 | 17.25 | 93.34 |

**Supplementary Table 6.** Post-hoc power analysis for the Landform main effect in the two-way ANOVA.

| **Outcome** | **F (1,120)** | ***P* value** | **Partial η²** | **Post-hoc power** |
| --- | --- | --- | --- | --- |
| Palatable herbage biomass | 3.148 | 0.079 | 0.026 | 0.427 |
| Palatable shrub biomass | 0.015 | 0.904 | 0.000 | 0.052 |
| Palatable tree biomass | 14.218 | < 0.001 | 0.106 | 0.965 |
| Total palatable biomass | 7.505 | 0.007 | 0.059 | 0.782 |
| Unpalatable herbage biomass | 4.651 | 0.033 | 0.037 | 0.578 |
| Unpalatable shrub biomass | 16.500 | < 0.001 | 0.121 | 0.982 |
| Unpalatable tree biomass | 2.231 | 0.138 | 0.018 | 0.321 |
| Total unpalatable biomass | 12.708 | < 0.001 | 0.096 | 0.946 |
| Carrying capacity | 2.31 | 0.131 | 0.019 | 0.33 |

Post-hoc power was calculated at α = 0.05 from the observed F statistic and its associated degrees of freedom.
